# The *Campylobacter jejuni* helical to coccoid transition involves changes to peptidoglycan and the ability to elicit an immune response

**DOI:** 10.1111/mmi.14269

**Published:** 2019-05-20

**Authors:** Emilisa Frirdich, Jacob Biboy, Mark Pryjma, Jooeun Lee, Steven Huynh, Craig T. Parker, Stephen E. Girardin, Waldemar Vollmer, Erin C. Gaynor

**Affiliations:** ^1^ Department of Microbiology and Immunology University of British Columbia Vancouver BC Canada; ^2^ The Centre for Bacterial Cell Biology, Institute for Cell and Molecular Biosciences Newcastle University Newcastle upon Tyne United Kingdom; ^3^ Department of Laboratory Medicine and Pathobiology University of Toronto Toronto ON Canada; ^4^ Produce Safety and Microbiology Unit, Western Region Research Center USDA Agricultural Research Service Albany CA USA

## Abstract

*Campylobacter jejuni* is a prevalent enteric pathogen that changes morphology from helical to coccoid under unfavorable conditions. Bacterial peptidoglycan maintains cell shape. As *C. jejuni* transformed from helical to coccoid, peptidoglycan dipeptides increased and tri‐ and tetrapeptides decreased. The DL‐carboxypeptidase Pgp1 important for *C. jejuni* helical morphology and putative N‐acetylmuramoyl‐L‐alanyl amidase AmiA were both involved in the coccoid transition. Mutants in *pgp1* and *amiA* showed reduced coccoid formation, with ∆*pgp1*∆*amiA* producing minimal coccoids. Both ∆*amiA* and ∆*amiA*∆*pgp1* lacked flagella and formed unseparated chains of cells consistent with a role for AmiA in cell separation. All strains accumulated peptidoglycan dipeptides over time, but only strains capable of becoming coccoid displayed tripeptide changes. *C. jejuni* helical shape and corresponding peptidoglycan structure are important for pathogenesis‐related attributes. Concomitantly, changing to a coccoid morphology resulted in differences in pathogenic properties; coccoid *C. jejuni* were non‐motile and non‐infectious, with minimal adherence and invasion of epithelial cells and an inability to stimulate IL‐8. Coccoid peptidoglycan exhibited reduced activation of innate immune receptors Nod1 and Nod2 versus helical peptidoglycan. *C. jejuni* also transitioned to coccoid within epithelial cells, so the inability of the immune system to detect coccoid *C. jejuni* may be significant in its pathogenesis.

## Introduction

Bacteria come in a wide variety of shapes and sizes. Shape and size are generally conserved within a genus, and elaborate mechanisms exist to ensure that bacteria maintain their shape during growth and division, indicating that morphology provides selective advantages to different growth environments and affects the biology of the organism (Young, 2006; 2007). As part of their lifecycle or under unfavorable growth conditions, some bacteria are capable of changing shape, thus altering their biological properties (Young, 2006; Justice *et al.*, 2008; Frirdich and Gaynor, 2013).

The Gram‐negative bacterium *Campylobacter jejuni* is a highly motile, helical organism that is a leading cause of bacterial foodborne gastroenteritis worldwide. Natural reservoirs include the environment, such as water sources, and animals, particularly avian species (Dasti *et al.*, 2010; Kirkpatrick and Tribble, 2011; Epps *et al.*, 2013). This is despite fastidious growth requirements in the laboratory: *C. jejuni* is microaerophilic, capnophilic, thermophilic (requiring growth temperatures ranging from 37 to 42°C), and are limited in their ability to ferment or oxidize carbohydrates as a nutrient source (Silva *et al.*, 2011). *C. jejuni* disease outcome ranges from mild, self‐limiting to severe, bloody diarrhea and can result in serious sequelae including inflammatory bowel disease, reactive arthritis, and Guillain Barré syndrome (Kirkpatrick and Tribble, 2011; Nyati and Nyati, 2013). The morphology of *C. jejuni* is helical during exponential growth but transitions from a helical to a coccoid form in stationary phase and under stress conditions such as starvation, suboptimal temperatures, oxidative stress, and changes in pH and osmolarity, at rates that vary depending on the conditions (Svensson *et al.*, 2008; Jackson *et al.*, 2009; Ikeda and Karlyshev, 2012). The related helical organism *Helicobacter pylori* also undergoes a helical to coccoid morphological transition.

*C. jejuni* transformation to a coccoid form correlates with entrance into a viable but non‐culturable (VBNC) state. However, coccoid formation is not an exclusive requirement, as some helical *C. jejuni* cells can also be VBNC (Svensson *et al.*, 2008; Ikeda and Karlyshev, 2012). The VBNC state is an important survival mechanism for numerous non‐spore forming bacteria, including pathogens, and represents an inactive form of the bacteria allowing survival under adverse environmental conditions until bacterial growth and cell division can resume (Oliver, 2010; Ramamurthy *et al.*, 2014; Ayrapetyan *et al.*, 2015). Considerable debate exists as to whether the *C. jejuni* coccoid form is a dormant state or simply a degenerative form of the organism [reviewed in (Svensson *et al.*, 2008; Ikeda and Karlyshev, 2012)]. As first suggested by Hazeleger *et al.* (1995), a good explanation for variable results reported in the literature regarding the characteristics of coccoid *C. jejuni* is that there are different types of coccoid cells with different characteristics depending on the conditions under which the coccoid cells were formed. For example, coccoid *C. jejuni* cells formed at higher temperatures and in nutrient‐rich conditions display much more degeneration and a faster loss of culturability than those formed at lower temperatures and in an environment with low nutrient concentrations (Svensson *et al.*, 2008; Ikeda and Karlyshev, 2012).

The peptidoglycan (PG) sacculus is a mesh‐like layer surrounding the bacterial cytoplasmic membrane, providing strength and rigidity to the cell wall. It is composed of chains of alternating β1‐4 linked N‐acetylglucosamine (GlcNAc) and N‐acetylmuramic acid (MurNAc) residues that are connected through short peptides attached to the MurNAc residue. Cell shape is maintained in most bacteria by the PG sacculus. This is also true for *C. jejuni*, as deletion of PG hydrolases Pgp1 (Frirdich *et al.*, 2012) and Pgp2 (Frirdich *et al.*, 2014) alters the PG muropeptide profile, causing a loss of the helical shape and rod‐shaped morphology. Both enzymes are involved in PG post‐biosynthetic remodeling, trimming the PG peptide sidechain. Pgp1 is a DL‐carboxypeptidase cleaving PG tripeptides to dipeptides and Pgp2 is an LD‐carboxypeptidase cleaving tetrapeptides to tripeptides. The loss of either of these hydrolases results in changes in PG structure and shape that affect the ability of *C. jejuni* to survive stress and interact with the host (Frirdich *et al.*, 2012; 2014; Stahl *et al.*, 2016).

*C. jejuni* can adhere to, invade, and survive within epithelial cells; these attributes are used frequently as *in vitro* measures of virulence [reviewed in (Dasti *et al.*, 2010; Rubinchik *et al.*, 2012)]. *C. jejuni* also triggers innate immune responses resulting in the production of proinflammatory chemokines and cytokines as well as the neutrophil chemoattractant IL‐8 (van Putten *et al.*, 2009). Bacterial muropeptides can activate the cytoplasmic human nucleotide‐binding oligomerization domain (Nod) receptors (Zilbauer *et al.*, 2007; Al‐Sayeqh *et al.*, 2010). The Nod1 receptor preferentially recognizes diaminopimelic acid (DAP)‐containing tripeptides found in Gram‐negative bacterial PG, while Nod2 recognizes dipeptides from both Gram‐negative and Gram‐positive bacterial PG (Benko *et al.*, 2008; Boudreau *et al.*, 2012; Kumar *et al.*, 2012). Previous studies with ∆*pgp1* and ∆*pgp2* mutant muropeptides indicates that for *C. jejuni*, these paradigms hold for Nod1 with levels of tripeptides correlating to Nod1 activation (Frirdich *et al.*, 2012; 2014). This was not the case for Nod2. Nod2 activation levels did not vary from wild type, despite varying levels of dipeptides in ∆*pgp1* and ∆*pgp2* mutant muropeptides (Frirdich *et al.*, 2012; 2014).

Our previous work characterized the PG structure and biosynthesis of helical *C. jejuni* and the role of *C. jejuni* morphology on the biology of this organism (Frirdich *et al.*, 2012; 2014; 2017; Stahl *et al.*, 2016). This current study builds on that work by examining the PG muropeptide profile of coccoid *C. jejuni*, the genetic determinants involved in PG remodeling required to generate the coccoid form, and the effects of coccoid cells and PG on host cell interactions.

The *C. jejuni* PG muropeptide profile *as C. jejuni* transitions from a helical to a coccoid morphology showed that coccoid *C. jejuni* had an increase in PG dipeptides and a reduction in tripeptides and tetrapeptides. The *C. jejuni* Pgp1 DL‐carboxypeptidase, important for helical morphology, also played a partial role in remodeling PG during the transition from a helical to coccoid form. In *H. pylori*, a mutant in the PG hydrolase *amiA* gene was completely defective in the transition from a helical form to a coccoid one (Chaput *et al.*, 2006; Chaput *et al.*, 2016). AmiA is an N‐acetylmuramoyl‐L‐alanine amidase cleaving the PG muropeptide peptide sidechain and is important in septum cleavage during cell division. The *C. jejuni* genome encodes for one annotated and previously uncharacterized *amiA* gene. A *C. jejuni amiA* mutant formed long chains of unseparated cells indicative of a cell division defect and was also delayed in coccoid formation. A *C. jejuni* ∆*pgp1*∆*amiA* double mutant nearly completely abrogated coccoid formation.

Consistent with previous observations that *C. jejuni* morphology affects pathogenesis, epithelial cells were unresponsive to coccoid cells and did not trigger an inflammatory response: unlike helical cultures, coccoid cultures were defective for adherence, invasion and intracellular survival in epithelial cells, did not stimulate IL‐8 production, and coccoid PG and muropeptides triggered reduced Nod1 and Nod2 activation in comparison to helical PG. Indeed, the inability of the immune system to detect coccoid *C. jejuni* may be significant in the pathogenic cycle of *C. jejuni*, especially considering that wild type *C. jejuni* was shown to transition to a coccoid form within epithelial cells.

## Results

### *C. jejuni* 81‐176 transitions to a coccoid form during prolonged incubation/starvation

**Figure 1:**
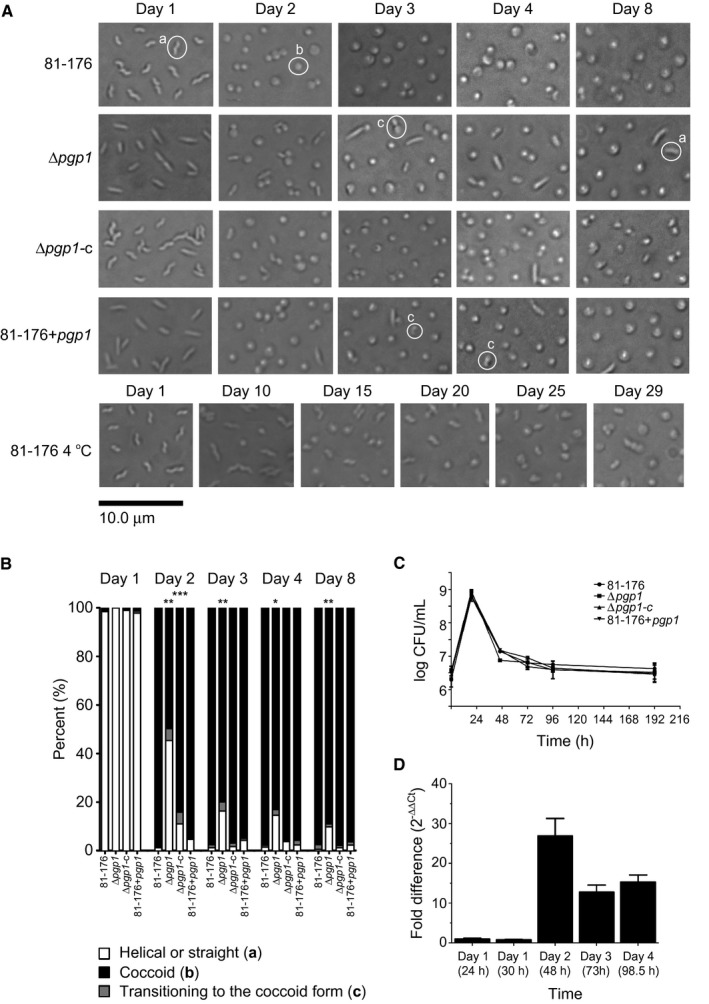
The *C. jejuni* 81‐176 transition from a helical to coccoid form was delayed in a ∆*pgp1* mutant and at 4°C in comparison to 38°C. A. DIC microscope images of *C. jejuni* wild type 81‐176, ∆*pgp1* mutant strain, ∆*pgp1* complemented strain (∆*pgp1‐*c), and *pgp1* overexpressing strain (81‐176 + *pgp1*) grown on solid media at 38°C or 4°C to follow the transition to the coccoid form over time. Representative cells considered to be helical, coccoid or transitioning to the coccoid form are indicated by a, b or c, respectively in the images. B. the percentage of helical, coccoid and cells transitioning to the coccoid form as determined from DIC images such as those shown in A. At least three separate fields of view of approximately 200 bacteria/field of view were counted for each strain at each timepoint and this was carried out in triplicate. The asterisk (*) indicates a statistically significant difference using the unpaired Student's t‐test as compared to the wild type 81‐176 with *, ** or *** indicating *p* < 0.05, *p* < 0.01 and *p* < 0.0001, respectively. C. growth curve analysis of *C. jejuni* wild type 81‐176, ∆*pgp1*, ∆*pgp1‐*c and 81‐176 + *pgp1* grown in broth. D. expression of the *pgp1* gene was monitored during growth on solid media at 38°C over time by RT‐qPCR. Bars represent the fold difference relative to Day 1 at 24 h. Data are representative of three independent experiments.

Several environmental stresses result in *C. jejuni* coccoid formation, including starvation, suboptimal temperatures, changes in oxygen tension, pH, osmolarity and pressure [reviewed in (Svensson *et al.*, 2008; Ikeda and Karlyshev, 2012)]. For ease of analysis, prolonged growth/starvation was used in this study for coccoid formation. Cells were grown at 38°C on solid media (Fig. 1A, wild type strain 81‐176) and in liquid media (data not shown) under microaerophilic conditions, and coccoid formation was monitored by microscopy over time. All samples were taken from the center of the agar plate, as sampling from different areas of the plate showed some variability. The percentage of helical, coccoid, and cells transitioning to the coccoid form was quantified from differential interference contrast (DIC) images (Fig. 1B, wild type strain 81‐176; Table S1). After 2 days post‐inoculation, approximately 98% of the population had transformed from the helical to coccoid form on solid media. In broth culture, the coccoid transition was slightly slower and more variable: after growth in liquid media for 4 days, approximately 94% of the population transformed to the coccoid form (Table S1). However, the growth curves (measured in CFU) for *C. jejuni* 81‐176 grown in broth and on solid media were identical (data not shown). The percentage of coccoid bacteria in the wild type 81‐176 strain in both solid and liquid media remained stable after the initial 4 days of growth (Fig. 1A and B, Table S1 & data not shown). Cultures grown for 4 days were selected as a source of coccoid bacteria for further analyses, as that was the time point at which both solid and liquid grown bacteria had reached a maximum level of coccoid formation. When possible, plate grown bacteria were used as the coccoid transition was more uniform and allowed for growth of a higher density of bacteria required for PG analysis.

For comparison to *C. jejuni* coccoids formed by prolonged growth at 38°C under microaerophilic conditions, coccoid *C. jejuni* formed by aerobic incubation at 4°C were also examined. *C. jejuni* cells grown for 1 day at 38°C under microaerophilic conditions were incubated aerobically at 4°C. After 29 days at 4°C, the bacterial population was no longer culturable, and 89% of the cells displayed a coccoid morphology which had been consistent over the previous 7 days (Fig. 1A). A larger proportion displayed a transitional state than bacteria grown for 4 days at 38°C (Table S1).

### Pgp1 affects coccoid formation

The coccoid transition of a ∆*pgp1* mutant was examined over time (Fig. 1A and B, Table S1). After growth for 2 days on solid media, the ∆*pgp1* mutant population was 50% coccoid in comparison to 98% for the wild type. Over time, the percentage of coccoid cells in the ∆*pgp1* mutant population increased to 80, 83 and 89% after 3, 4 and 8 days, respectively. The level of coccoid cells remained at 89% even after 12 days (data not shown). The ∆*pgp1* complemented strain (∆*pgp1‐c*) partially complemented the shape transition defect, with the percentage of coccoid cells in the population being 84% at day 2 and reaching near wild‐type levels of 97% by day 3 (Fig. 1A and B, Table S1). A *pgp1* overexpressing strain (81‐176 + *pgp1*) exhibiting cells with a straight or kinked morphology along with helical cells had an altered PG muropeptide structure in comparison to wild type (Frirdich *et al.*, 2012). This strain showed no statistically significant defect in coccoid transition. Despite differences in coccoid transition, the wild type 81‐176, ∆*pgp1* mutant, the ∆*pgp1* complemented strain, and the *pgp1* overexpressing strain (81‐176 + *pgp1*) all showed similar long‐term growth profiles (Fig. 1C). The LD‐carboxypeptidase encoded by *C. jejuni pgp2* cleaves tetra‐ to tripeptides and is also required for helical morphology (Frirdich *et al.*, 2014). Like ∆*pgp1*, ∆*pgp2* is rod‐shaped, although its PG muropetide profile is distinct from that of ∆*pgp1* (Frirdich *et al.*, 2012; Frirdich *et al.*, 2014). The ∆*pgp2* mutant displayed wild‐type coccoid formation timelines (Fig. S1, Table S1), as did the ∆*pgp1*∆*pgp2* double mutant (Fig. S1, Table S1) that phenotypically resembles a ∆*pgp2* mutant (Frirdich *et al.*, 2014).

Expression of the *pgp1* gene in the wild‐type 81‐176 strain was tracked over 4 days by RT‐qPCR (Fig. 1D). Expression of *pgp1* was unchanged at 24 h and 30 h and increased 26.9‐fold at 48 h (Day 2), corresponding to the timepoint at which the morphology of the population was coccoid. The levels of *pgp1* expression dropped at Day 3 and Day 4, but remained higher than at Day 1 (24 h). This is supported by differential expression analysis of RNAseq (high throughput sequencing of cDNA libraries) data showing that the *pgp1* gene (*cjj81‐176_1344*) transcript increased in abundance *in vitro* in stationary phase in comparison to mid‐log cultures (Taveirne *et al.*, 2013).

### *C. jejuni* AmiA affects coccoid formation

#### Bioinformatic analyses

In *H. pylori*, a mutant in the PG hydrolase *amiA* was defective in the transition from a helical form to a coccoid (Chaput *et al.*, 2006; Chaput *et al.*, 2016). The *C. jejuni amiA* gene had not been previously characterized, but we hypothesized it might also be involved in coccoid formation. As in *H. pylori*, the *C. jejuni* genome encodes for only one annotated N‐acetylmuramoyl‐L‐alanine amidase (Cjj81176_1285; AmiA). The *C. jejuni* 81‐176 and *H. pylori* 26695 amidases share 55% identity/70% similarity in their C‐terminal domains (amino acids 426‐658 of the *C. jejuni* AmiA) that encodes for the highly conserved amidase_3 (pfam01520) domain containing the catalytic site (Fig. S2). They also have two smaller regions of identity at the N‐terminus: 21% identity/40% similarity in *C. jejuni* amino acids 202–313 and 38% identity/61% similarity in *C. jejuni* amino acids 346–395. The *E. coli* genome encodes for five N‐acetylmuramoyl‐L‐alanine amidases: the periplasmic amidases AmiA, AmiB, AmiC, AmiD and the cytoplasmic AmpD. AmiA, AmiB and AmiC belong to the same family of amidases as AmiA from *H. pylori* and *C. jejuni*. The *C. jejuni* AmiA at 659 amino acids is longer than the other amidases (*H. plyori* AmiA – 440 amino acids*; E. coli* AmiA – 289 amino acids, AmiB ‐ 445 amino acids and AmiC – 417 amino acids), but conserved domain searches do not detect any additional domains in *C. jejuni* AmiA. Neither *C. jejuni* nor *H. pylori* AmiA proteins show a conserved AMIN domain (pfam11741) found in *E. coli* AmiC thought to be involved in periplasmic targeting and protein localization.

#### ∆*amiA* is defective in cell separation after division and lacks flagella

**Figure 2:**
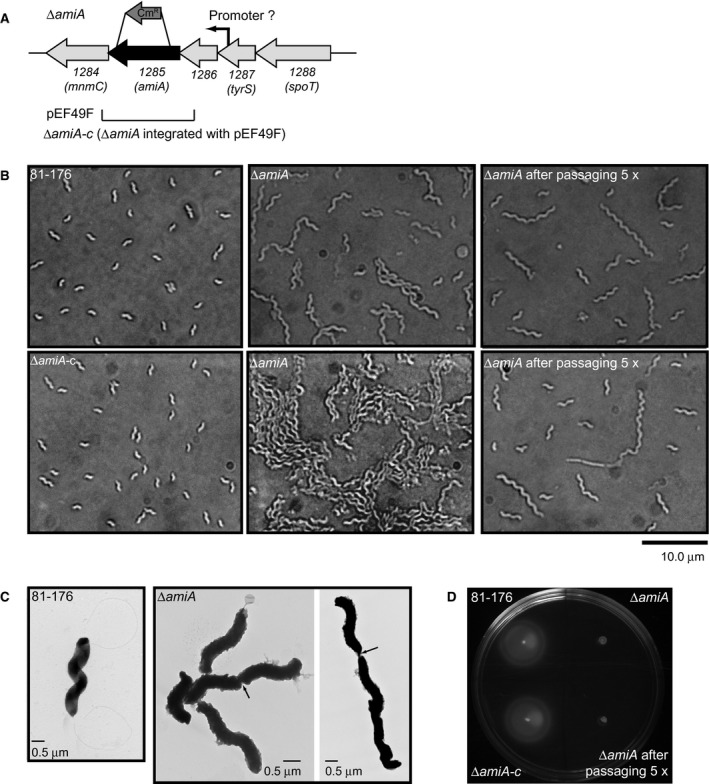
*C. jejuni* 81‐176 *amiA* gene locus, ∆*amiA* mutant morphology and motility defect. A. The ∆*amiA* mutant was constructed by deleting a 1537 bp internal fragment of the 1980 bp *amiA* gene; the approximate location of this deletion is shown above the gene cluster and is denoted by the ∆*amiA* strain designation. The region cloned into the integrative vector pRRK (pEF49F; Km^R^) used for complementation is shown below the gene cluster. The putative promoter predicted by RNA‐seq is indicated (Dugar et al., 2013). B. DIC microscope images of the helical *C. jejuni* 81‐176 strain, the ∆*amiA* mutant strain showing chains of pleomorphic cell lengths (short chains in the top center panel and long clumps of chains in the bottom center panel)*,* a lab passaged ∆*amiA* mutant strain with a decreased number of long chained cells (top and bottom panel), and complemented strain ∆*amiA‐c* in which the cell division defect has been restored. C. negatively stained TEM images of the helical *C. jejuni* 81‐176 wild‐type strain with intact flagella and the ∆*amiA* mutant strain lacking flagella with arrows indicating the presence of fully formed septa. D. ∆*amiA* is non‐motile in soft agar plates, as expected from its lack of flagella. The non‐motile phenotype is retained after lab passaging. Wild‐type motility is restored by complementation.

A deletion mutant in *amiA* was constructed to determine whether the *C. jejuni amiA* gene plays a similar role in the transition to the coccoid form as the *H. pylori* homolog (Chaput *et al.*, 2006; Chaput *et al.*, 2016) (Fig. 2A). The ∆*amiA* mutant was very slow growing, and the colonies were extremely tacky and difficult to pass. With passage, the growth rate of the mutant increased and the consistency of the colonies returned to that of wild type. After 24 h growth on plates (the ∆*amiA* mutant did not grow in broth), the cell morphology of the ∆*amiA* mutant population was observed to be composed of helical chains of cells of pleomorphic lengths from shorter chain (Fig. 2B, ∆*amiA* top panel) to extremely long chains of cells that clumped together (Fig. 2B, ∆*amiA* lower panel). Difficulties associated with clumping of the cells during sample processing also precluded capturing transmission electron microscopy (TEM) images of the long chains. TEM images of shorter chains showed the presence of a fully formed septum, indicating that the mutant had a cell separation defect and a lack of flagella (Fig. 2C). The outer membrane showed extensive evidence of bulging and blebbing. As expected from the lack of flagella, the ∆*amiA* mutant was completely non‐motile in soft agar plates (Fig. 2D). After 5 × passage, the population of ∆*amiA* mutant cells consisted of short chains of cells (Fig. 2B top and bottom panel) and had lost the portion of the population consisting of very long chains. Some straight cells were present among the helical cells (Fig. 2B bottom panel). This was also evident in the minimally passaged ∆*amiA* population. The passaged ∆*amiA* mutant remained non‐motile in soft agar plates and likely had not regained the ability to form flagella (Fig. 2D). No genomic changes unique to passaged ∆*amiA* mutant strains (EF262‐P5 and EF280‐P5, both passaged 5 times, and EF188 with an unknown amount of passages) indicative of suppressor mutations were detected by whole genome sequencing (Table S2, methods described in Supplemental Experimental Procedures). Complementation of ∆*amiA* restored the growth, cell separation and motility defects (Fig. 2A, B, D).

#### ∆*amiA* and ∆*amiA*∆*pgp1* are defective for coccoid formation

**Figure 3:**
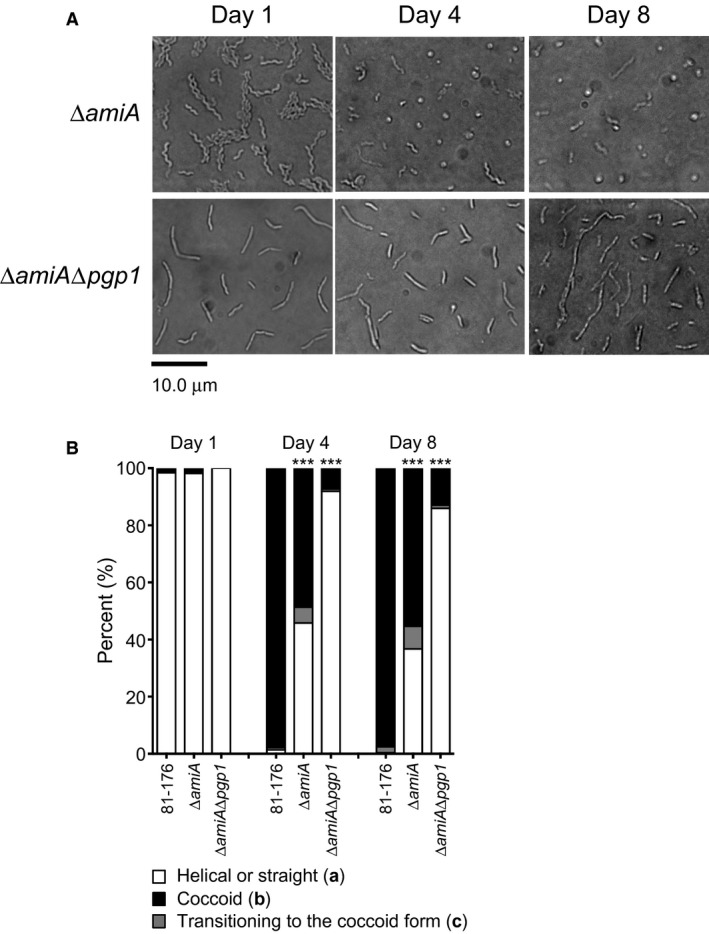
A *C. jejuni* 81‐176 ∆*amiA* mutant was delayed in coccoid formation, while a ∆*amiA*∆*pgp1* was nearly completely unable to form coccoid cells. A. DIC microscope images of the *C. jejuni* ∆*amiA* and ∆*amiA*∆*pgp1* mutant strains were taken over 8 days of growth at 38°C on solid media to follow the transition to the coccoid form over time. These can be compared to those of the wild type shown in Fig. 1A. At least three separate fields of view of approximately 200 bacteria/field of view were counted for each strain at each timepoint, and this was carried out in triplicate. B. the percentage of helical, coccoid and cells transitioning to the coccoid form as determined from DIC images such as those shown in A. The asterisk (*) indicates a statistically significant difference using the unpaired Student's t‐test as compared to the wild type 81‐176 with *** indicating *p* < 0.0001.

Transition of the ∆*amiA* mutant to the coccoid form during growth at 38°C on solid media under microaerobic conditions was tracked by microscopy (Fig. 3A). The percentage of bacteria with a coccoid morphology was determined from the DIC images (Fig. 3B). After 4 days of growth, 48.6% of the ∆*amiA* population displayed a coccoid morphology, in contrast to 97.8% of the wild type. By 8 days, this number increased to 55.3%.

With *C. jejuni* ∆*pgp1* and ∆*amiA* single mutants being delayed and defective for the coccoid transition, respectively, a ∆*amiA*∆*pgp1* double mutant was generated to determine whether the defects in coccoid formation were cumulative. The coccoid transition of the ∆*amiA*∆*pgp1* double mutant was examined by microscopy, and cells displaying straight, coccoid and transitioning morphologies were quantified (Fig. 3B and Table S1). The ∆*pgp1*∆*amiA* mutant was highly defective for coccoid formation, with only 7.3% and 13.0% of the population displaying a coccoid morphology after growth for 4 and 8 days, respectively. This is in contrast to the day 8 ∆*pgp1* and ∆*amiA* populations being 89.0% and 55.3% coccoid, respectively (Table S1).

### Peptidoglycan from coccoid cells has an abundance of dipeptides

**Figure 4:**
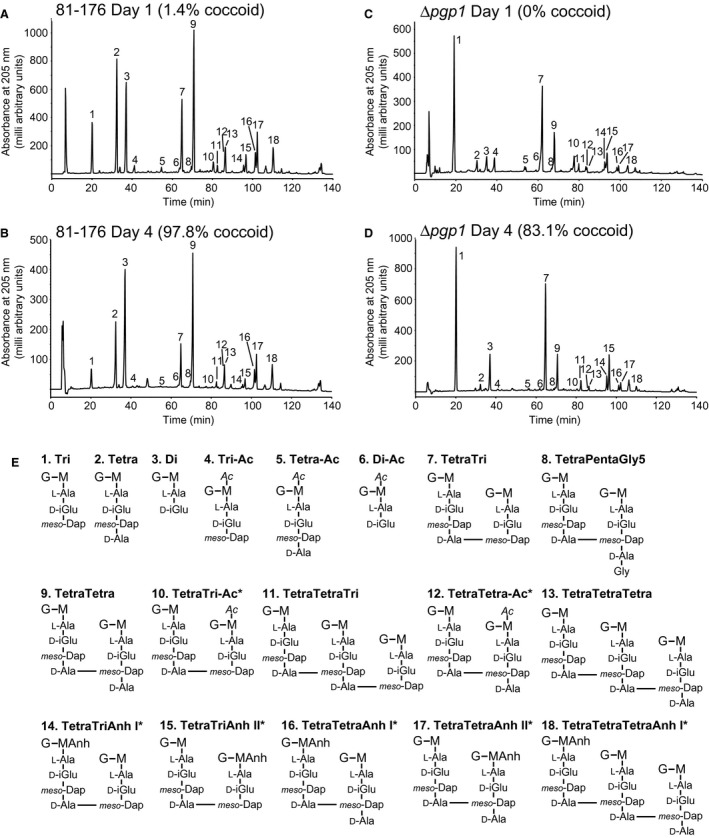
HPLC elution profile of *C. jejuni* wild type and ∆*pgp1* muropeptides and proposed muropeptide structures. Purified PG was digested with cellosyl, and the resulting muropeptides were reduced with sodium borohydride and separated on a Prontosil 120‐3‐C18 AQ reverse‐phase column. HPLC profiles are shown for A,* C. jejuni* wild‐type 81‐176 strain grown for 1 day demonstrating helical morphology. B. 81‐176 grown for 4 days demonstrating primarily coccoid morphology. C. the ∆*pgp1* mutant strain grown for 1 day demonstrating a rod‐shaped morphology. D. the ∆*pgp1* mutant strain grown for 4 days demonstrating a mixed rod (14.5%) and coccoid (83.1%) morphology. Peak numbers correspond to the main muropeptide peak fractions of *C. jejuni* 81‐176 analyzed by LTQ‐FT‐MS (Frirdich et al., 2012) to determine the structures shown in E. G. N‐acetylglucosamine; M, reduced N‐acetylmuramic acid; L‐Ala, L‐alanine; D‐iGlu, D‐isoglutamic acid; D‐Glu, D‐glutamic acid; *meso‐*DAP, *meso‐*diaminopimelic acid; Gly. Glycine; Ac, O‐acetyl groups at the C‐6 hydroxyl group of MurNAc; Anh, 1,6‐anhydro group at MurNAc. The asterisk (*) indicates that it is not known on which MurNAc residue the modification occurs.

Morphology is maintained by the PG layer, so it was predicted that a transition from a helical to a coccoid form may be accompanied by a change in PG structure. The muropeptide profile from 81‐176 and ∆*pgp1* PG isolated from a coccoid population of cells formed by growth for 4 days under microaerophilic conditions at 38°C was analyzed by HPLC and compared to that of a helical population grown for 1 day under similar conditions (Fig. 4 and Table 1). In the *C. jejuni* 81‐176 day 4 coccoid profile, the most striking and consistent changes in muropeptides between replicates were the increased amounts of dipeptides and reduced total tripeptides and monomeric tetrapeptides (Fig. 4B and Table 1). No change in tripeptides was detected in the ∆*pgp1* day 4 muropeptides (Fig. 4D and Table 1). Neither sample showed changes in cross‐linking. Since tripeptides did not vary in the ∆*pgp1* day 4 muropeptides, the PG muropeptide profile at day 4 was examined in strains with differences in tripeptide levels at day 1 in comparison to wild type: ∆*pgp2* that has no tripeptides at day 1 [Table 1; (Frirdich *et al.*, 2014)] but displays a wild‐type coccoid transition and is coccoid by day 4 (Fig. S1 and Table S1), and 81‐176 overexpressing *pgp1* (81‐176 + *pgp1*, Table 1), which shows increased processing of tripeptides at day 1 (high dipeptides and low tripeptides in comparison to wild type [Table 1, (Frirdich *et al.*, 2012)] but wild‐type levels of coccoid cells by day 4 (despite a slight delay in coccoid transition at earlier timepoints; Fig. 1A and B). By day 4, both ∆*pgp2* and 81‐176 + *pgp1* muropeptides showed increased dipeptides and decreased tetrapeptides similar to wild type (Table 1). Monomeric tripeptides did vary in these strains, with an increase to detectable levels in ∆*pgp2* and a decrease in 81‐176 + *pgp1* (Table 1).

**Table 1:** Summary of the muropeptide composition of the wild‐type 81‐176, ∆*pgp1*, 81‐176 + *pgp1* (*pgp1* overexpressing strain), and ∆*pgp2* strains grown for either 1 or 4 days at 38°C (strains were grown at 38°C unless otherwise indicated) or 29 days at 4°C.^a^

	*C. jejuni* strains												
	81‐176	81‐176	81‐176	81‐176	81‐176	81‐176 4°C	81‐176 4°C	∆*pgp1*	∆*pgp1*	∆*pgp2*	∆*pgp2*	81‐176 + *pgp1*	81‐176 + *pgp1*
	Day 1	Day 4	Day 1	Day 4	Day 1	Day 29	Day 29	Day 1	Day 4	Day 1	Day 4	Day 1	Day 4
**Shape**	Helical	Coccoid	Helical	Coccoid	Helical	Coccoid	Coccoid	Rod	Coccoid, few rods	Rod	Coccoid	Curved rod	Coccoid
**Strain designation** b	B‐1	B‐2	F‐1	F‐2	E‐1	E‐2	E‐2	A‐1	B‐3	B‐4	G‐1	A‐2	G‐2
**Strain used for comparison**		B‐1		F‐1		E‐1	B‐2		A‐1		B‐4		A‐2
**Muropeptide species**	**% Peak area**												
Monomers (Total)	41.6	42.3	42.2	41.9	43.5	44.9	44.9	43.4	45.9	40.8	40.8	42.1	44.1
Di	15.3	25.9*	13.6	25.3*	17.8	21.9*	21.9	5.9	9.1*	9.1	19.1*	18.4	31.9*
Tri	8.9	4.2*	10.1	4.4*	6.7	5.5	5.5*	34.4	35.4	0.0	0.8*	2.3	1.4*
Tetra	17.4	12.2*	17.2	11.6*	18.3	16.9	16.9*	3.1	1.4*	31.7	20.0*	21.5	10.0*
Penta	0.0	0.0	nd^4^	nd	0.0	0.3*	0.3*	0.0	0.0	nd	nd	nd	nd
PentaGly5	0.0	0.0	0.9	0.5*	0.6	0.3*	0.3*	0.0	0.0	nd	0.9	nd	0.7
Acetylated^c^	2.9	0.6	1.9	1.1	1.0	1.5	1.5	5.1	0.5	1.6	0.8	4.0	0.9
Dimers (Total)	47.9	45.4	48.8	46.1	47.7	46.6	46.6	52.5	49.3	47.6	46.6	50.0	41.3
TetraTri	16.0	10.2*	16.0	11.0*	16.8	15.9	15.9*	37.0	38.2	0.0	0.4*	3.7	4.0
TetraTetra	31.1	34.2	32.1	34.3	30.5	30.0	30.0	14.7	10.6*	46.8	44.7	44.4	35.5*
TetraPenta	0.0	0.0	nd	nd	0.0	0.2*	0.2*	0.0	0.0	nd	1.5	1.9	1.9
TetraPentaGly5	0.7	1.0*	0.8	0.8	0.4	0.5*	0.5*	0.7	0.5*	0.8	nd	nd	nd
Anhydro	12.5	12.5	11.8	13.0	13.5	11.8	11.8	11.7	14.9*	12.1	13.1	10.5	9.8
Acetylated^c^	2.4	0.4	3.2	1.2	2.2	1.8	1.8	6.8	0.5	0.5	0.0	5.5	0.3
Trimers (Total)	10.5	12.3	7.8	10.8*	8.8	8.5	8.5*	4.2	4.8*	11.6	12.7	7.9	13.7*
TetraTetraTri	1.0	1.2	1.0	1.1	0.9	0.8	0.8*	1.9	2.3*	0.0	0.4*	0.3	0.7*
TetraTetraTetra	9.5	11.1	6.8	9.8*	8.0	7.7	7.7*	2.3	2.5	11.6	12.2	7.6	13.1*
Dipeptides (Total)	15.3	25.9*	13.6	25.3*	17.8	21.9*	21.9	5.9	9.1*	9.1	19.1*	18.4	31.9*
Tripeptides (Total)	17.3	9.7*	18.4	10.3*	15.4	13.7	13.7*	53.6	55.2	0.0	1.1*	4.2	3.6
Tetrapeptides (Total)	67.0	63.9	65.1	62.3	66.0	63.3	63.3	40.2	35.4	90.5	78.1	76.5	61.9
Pentapeptides (Total)	0.4	0.5*	1.2	**0.9**	0.8	**1.0**	**1.0**	0.4	**0.3**	0.4	0.8*	1.0	1.0
Acetylated (Total)^c^	4.1	0.9	3.5	1.7	2.1	2.4	2.4	0.8	0.7	1.8	0.8	6.7	1.1
Anhydro chain ends (Total)	7.9	8.4	7.7	8.9	8.4	7.1	7.1	6.4	7.9*	8.0	8.9	6.9	7.7
Average chain length	12.7	11.9	13.0	11.2	12.0	14.1	14.1	15.7	**12.6**	12.5	**11.2**	14.5	12.9
Degree of cross‐linkage	31.0	30.9	29.6	30.3	29.7	29.0	29.0	29.0	27.9	31.6	31.7	30.2	29.8
% Peptides in cross‐links	58.4	57.7	57.8	58.2	56.5	55.1	55.1	56.6	54.1	59.2	59.3	57.9	56.0

nd = not determined.

a Numbers represent the percent area of each muropeptide from Table S4 calculated to give a total of 100%. Values indicated with an asterisk (*) or both bolded and with an asterisk, represent a greater than or equal to 20% or 30% difference, respectively in comparison to *C. jejuni* strains indicated.

b The strain designation is included to clarify strain‐to‐strain comparisons. It consists of a letter (A‐G) denoting the series in which the sample was analyzed ordered by date analyzed followed by a number denoting the sample within the series (1‐4). Samples analyzed in the same batch will have identical letters. Wherever possible, strains were compared to wild type from the same sample series. See Table S3 for further clarification of the series designation.

c The values for the percentage of O‐acetylated species do not represent the true level of O‐acetylation in these strains, as most of these substitutions are lost in the standard procedure used in this study to prepare and analyze the PG.

The rate of formation and the characteristics of coccoid cells vary depending on the coccoid‐inducing condition (Ikeda and Karlyshev, 2012). To determine whether the PG structure of coccoid *C. jejuni* cells formed under different conditions varied, PG was isolated from *C. jejuni* coccoid cells induced as a result of temperature and oxidative stress by incubating plates of *C. jejuni* aerobically at 4°C until the population reached a maximum amount of coccoid bacteria (not shown). The muropeptide profile was compared to that of the coccoid bacteria produced by incubation for 4 days at 38°C under microaerophilic conditions and showed a similar increase in the amount of monomeric dipeptides and reduced amounts of tri‐ and tetrapeptides, although the changes were not as large (Table 1). The aerobic/cold‐stressed coccoid cells did show some muropeptide changes that were not apparent in the starved coccoid cells: the presence of pentapeptides that had not previously been detected in *C. jejuni* muropeptide preparations, as well as a slight increase in average chain length indicated by a reduction in the amount of anhydropeptides.

### Peptidoglycan from an ∆*amiA* mutant and ∆*amiA*∆*pgp1*

**Figure 5:**
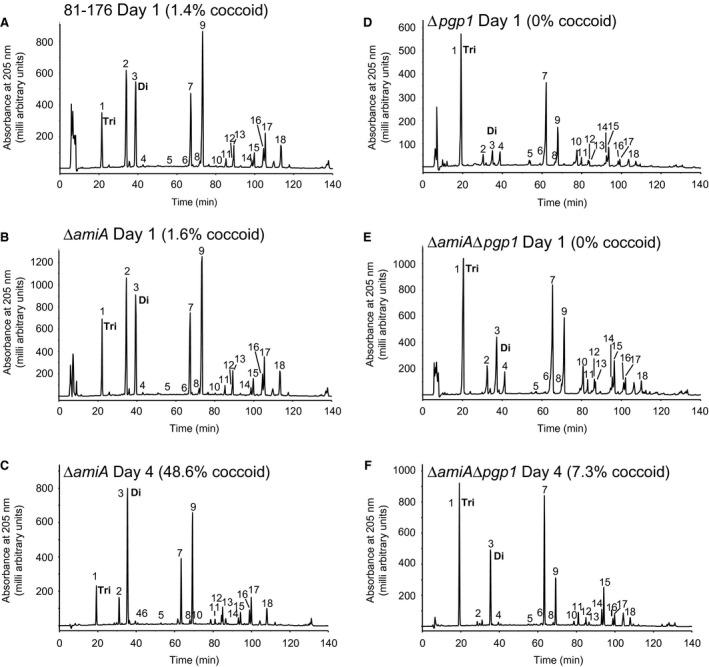
HPLC elution profile of *C. jejuni* 81‐176 wild type, ∆*amiA,* ∆*pgp1,* and ∆*amiA* ∆*pgp1* muropeptides. Purified PG from strains was digested with cellosyl and the resulting muropeptides were reduced with sodium borohydride and separated on a Prontosil 120‐3‐C18 AQ reverse‐phase column. HPLC profiles are shown for A,* C. jejuni* wild‐type 81‐176 strain grown for 1 day demonstrating helical morphology; B, the ∆*amiA* mutant strain grown for 1 day  demonstrating a helical morphology in long chains of unseparated cells; C, the ∆*amiA* mutant strain grown for 4 days with a population morphology of partially coccoid cells and some helical chains of cells; D, the ∆*pgp1* mutant strain grown for 1 day demonstrating a rod‐shaped morphology; E, the ∆*amiA*∆*pgp1* mutant strain grown for 1 day with a morphology consisting of straight chains of cells and F, the ∆*amiA*∆*pgp1* mutant strain grown for 4 days with a morphology consisting of straight chains of cells. Peak numbers correspond to the main muropeptide peak fractions of *C. jejuni* 81‐176 analyzed by LTQ‐FT‐MS (Frirdich et al., 2012) and shown in Fig. 4E. The Tri (peak 1) and Di (peak 3) species are indicated on the muropeptide profiles for comparison.

The muropeptide profile of a *C. jejuni* ∆*amiA* mutant was nearly unchanged in comparison to wild type at day 1 (Fig. 5 and Table 2), even in repeated analyses (data not shown). Overexpression of *amiA* resulted in an increase in dipeptides. After 4 days of growth, the ∆*amiA* mutant was partially coccoid, and the muropeptide profile showed an increase in dipeptides (unlike *H. pylori* ∆*amiA* that accumulated much lower levels of dipeptides) and a decrease in tetrapeptides in comparison to the same strain at day 1 (Fig. 5 and Table 2). No significant change in tripeptides was detected. Despite 81‐176 and ∆*amiA* muropeptides having a similar profile at day 1, by day 4 ∆*amiA* had lower tetrapeptides, and higher tripeptides than 81‐176 at day 4 (Fig. 5 and Table 2).

**Table 2:** Summary of the muropeptide composition of the *C. jejuni* wild‐type 81‐176, ∆*amiA* and ∆*amiA*∆*pgp1* mutant strains grown for 1 or 4 days at 38°C.^a^

	*C. jejuni* strains												
	81‐176	∆*amiA*	81‐176	81‐176 + *amiA*	∆*pgp1*	∆*amiA*∆*pgp1*		81‐176	∆*amiA*	∆*amiA*	∆*amiA*∆*pgp1*	∆*amiA*∆*pgp1*	∆*pgp1*
	Day 1	Day 1^b^	Day 1	Day 1	Day 1	Day 1		Day 4	Day 4	Day 4	Day 4	Day 4	Day 4
**Shape**	Helical	Helical chains	Helical	Helical	Rod	Straight chains		Coccoid	Coccoid, few chains	Coccoid, few chains	Straight chains	Straight chains	Coccoid, few rods
**Strain designation** c	D‐1	D‐2	F‐1	F‐4	A‐1	C‐1		F‐2	F‐3	F‐3	F‐5	F‐5	B‐3
**Strain used for comparison**		D‐1		F‐1		A‐1		F‐1	F‐2	D‐2	C‐1	B‐3	
*Muropeptide species*	*% Peak area*												
Monomers (Total)	41.2	41.9	42.2	43.0	43.4	44.0	42.2	41.9	43.4	43.4	42.8	42.8	45.9
Di	16.1	15.3	13.6	17.0*	5.9	11.4*	13.6	25.3*	30.4*	30.4*	17.0*	17.0*	9.1
Tri	8.4	9.1	10.1	8.5	34.4	28.2	10.1	4.4*	7.6*	7.6	24.8	24.8*	35.4
Tetra	16.7	17.5	17.2	16.1	3.1	4.4*	17.2	11.6*	5.4*	5.4*	0.9*	0.9*	1.4
PentaGly5	nd^5^	nd	0.8	1.0*	nd	nd	0.8	0.5*	0.0*	nd	0.0	0.0	0.0
Acetylated^d^	0.6	0.6	1.9	3.3	5.1	5.4	1.9	1.1	2.1	2.1	1.4	1.4	0.5
Dimers (Total)	49.5	49.1	48.8	47.7	52.5	50.5	48.8	46.1	47.5	47.5	48.0	48.0	49.3
TetraTri	15.7	15.9	16.0	14.9	37.0	31.7	16.0	11.0*	16.3*	16.3	34.8	34.8	38.2
TetraTetra	33.1	32.5	32.0	31.7	14.7	17.5	32.0	34.3	30.4	30.4	12.7*	12.7	10.6
TetraPentaGly5	0.8	0.7	0.8	1.2*	0.7	1.4*	0.8	0.8	0.8	0.8	0.5*	0.5	0.5
Anhydro	12.0	12.0	11.8	11.9	11.7	10.5	11.8	13.0	11.7	11.7	13.0*	13.0	14.8
Acetylated^d^	0.3	0.3	3.2	4.0	6.8	7.1	3.2	1.2	2.7	2.7	1.3	1.3	0.5
Trimers (Total)	9.3	9.0	7.8	8.2	4.2	5.5*	7.8	10.8*	8.1*	8.1	5.6	5.6	4.8
TetraTetraTri	1.2	1.2	1.0	1.3*	1.9	1.9	1.0	1.1	1.2	1.2	2.4*	2.4	2.3
TetraTetraTetra	8.1	7.8	6.8	6.8	2.3	3.7*	6.8	9.8*	6.9*	6.9	3.2	3.2*	2.5
Dipeptides (Total)	16.1	15.3	13.6	17.0*	5.9	11.4*	13.6	25.3*	30.4*	30.4*	17.0*	17.0*	9.1
Tripeptides (Total)	16.6	17.4	18.4	16.4	53.6	44.6	18.4	10.3*	16.1*	16.1	43.0	43.0*	55.2
Tetrapeptides (Total)	66.9	66.9	65.1	63.5	40.2	43.3	65.1	62.3	52.1	52.1*	36.1	36.1	35.4
Pentapeptides (Total)	0.4	0.4	1.2	1.6*	0.4	0.7*	1.2	**0.9**	0.4	0.4	0.2*	0.2*	0.3
Acetylated (Total)^d^	0.8	0.7	3.5	5.3*	8.5	8.9	3.5	1.7	3.5	3.5	2.0	2.0	0.7
Anhydro chain ends (Total)	7.6	7.5	7.7	7.7	6.4	6.1	7.7	8.9	7.6	7.6	8.4*	8.4	7.9
Average chain length	13.2	13.3	13.0	12.9	15.7	16.4	13.0	11.2	13.2	13.2	11.9*	11.9	12.6
Degree of cross‐linkage	31.0	30.5	29.6	29.3	29.0	28.9	29.6	30.3	29.1	29.1	27.7	27.7	27.8
% Peptides in cross‐links	58.8	58.1	57.8	57.0	56.6	56.0	57.8	58.2	56.6	56.6	57.2	57.2	54.1

nd = not determined.

a Numbers represent the percent area of each muropeptide from Table S4 calculated to give a total of 100%. Values indicated with an asterisk (*) or both bolded and with an asterisk, represent a greater than or equal to 20% or 30% difference, respectively in comparison to *C. jejuni* strains indicated.

b This experiment was repeated and showed similar results.

c The strain designation is included to clarify strain‐to‐strain comparisons. It consists of a letter (A–G) denoting the series in which the sample was analyzed ordered by date analyzed followed by a number denoting the sample within the series (1–4). Samples analyzed in the same batch will have identical letters. Wherever possible, strains were compared to wild type from the same sample series. See Table S3 for further clarification of the series designation.

d The values for the percentage of O‐acetylated species do not represent the true level of O‐acetylation in these strains, as most of these substitutions are lost in the standard alkaline reduction procedure used in this study to prepare the PG.

Many of the muropeptide changes in ∆*amiA*∆*pgp1* result from the deletion of *pgp1* [(Frirdich *et al.*, 2012); Fig. 5 and Table 2]. Changes in ∆*amiA*∆*pgp1* that appear to be unique to the deletion of *amiA* are higher dipeptides and monomeric tetrapeptides and an increase in trimers, in particular tetra‐tetra‐tetra trimers. Even though the level of pentapeptides appeared higher in ∆*amiA*∆*pgp1* than ∆*pgp1* at day 1, there were no significant changes in pentapeptides in comparison to the wild type strain analyzed along with ∆*amiA*∆*pgp1* (data not shown). The levels of muropeptides varied slightly with each preparation of a strain, with the percent differences appearing to be larger in species that are present in small amounts. After 4 days of growth, ∆*amiA*∆*pgp1* muropeptides showed an increase in dipeptides and a decrease in monomeric tetrapeptides in comparison to day 1 (Fig. 5 and Table 2). Similar to ∆*pgp1* and ∆*amiA* at day 4, there was no change in tripeptides in ∆*amiA*∆*pgp1*.

### *C. jejuni* wild type transitions to coccoid inside epithelial cells, which is delayed in ∆*pgp1*

**Figure 6:**
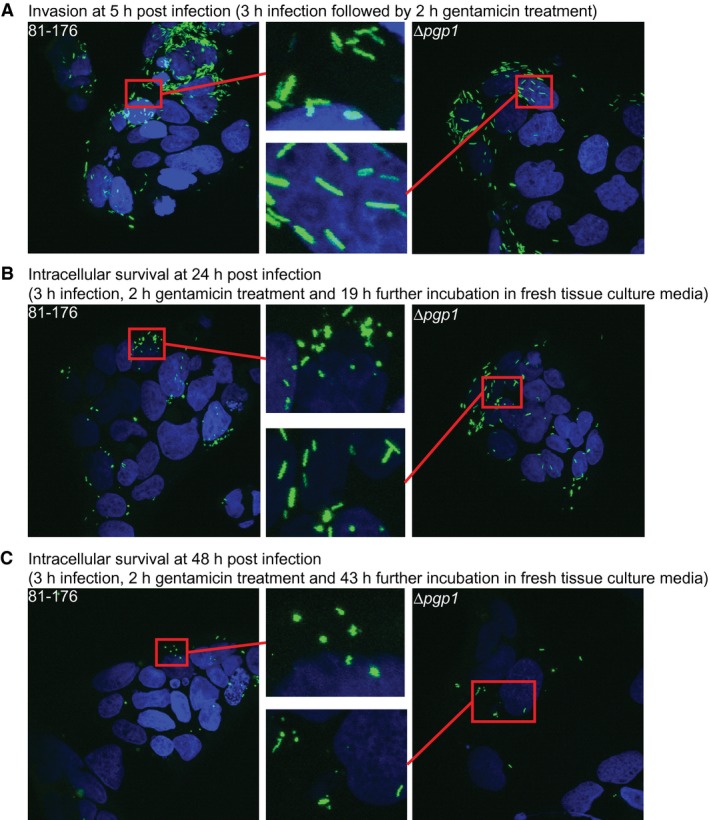
*C. jejuni* 81‐176 transitioned to a coccoid form inside epithelial cells which was delayed in a ∆*pgp1* mutant. INT407 epithelial cells were infected with the GFP‐expressing *C. jejuni* wild type 81‐176 (left panels) and ∆*pgp1* mutant (right panels) strains in a gentamicin (Gm) protection assay. After a 3 h infection period, the cells were treated for 2 h with Gm to kill the extracellular bacteria. After the Gm was washed off, the cells were incubated with fresh MEM containing 3% FBS and a low dose of Gm. Intracellular bacteria visualized 5 h, 24 h and 48 h post‐infection are shown in panels A, B, and C, respectively. INT407 cell nuclei were stained with DAPI and are shown in blue, while GFP‐expressing bacteria are shown in green. The area in each image highlighted by a red box is magnified (center panels). Three fields of view were visualized for each sample. Each experiment was performed in triplicate on duplicate samples of each strain.

To examine whether *C. jejuni* undergoes transformation to the coccoid form within epithelial cells, INT407 cells grown on coverslips were infected with helical, log‐phase *C. jejuni* 81‐176 expressing GFP from a plasmid in a gentamicin (Gm) protection assay. GFP‐expressing intracellular bacteria were visualized by confocal microscopy at 5, 24 and 48 h post‐infection (Fig. 6). At the 5 h timepoint, the wild‐type bacterial population appeared to be entirely helical (Fig. 6A). However, at 24 h (Fig. 6B) and even more so at 48 h (Fig. 6C), a large proportion of the population displayed a coccoid morphology. Due to bacterial cell clumping and the difficulties associated with differentiating the boundaries between individual bacterial cells, the proportion of helical to coccoid bacteria could not be quantified.

In an identical experiment to that described for wild type, a GFP‐expressing ∆*pgp1* strain was used to infect INT407 cells to determine whether the delay in coccoid transition seen with a ∆*pgp1* mutant in culture also occurred within epithelial cells. At the 5 h timepoint, the ∆*pgp1* mutant population was entirely straight with a few coccoid cells (Fig. 6A). At the 24 h timepoint, unlike wild type, which had nearly completely transitioned to a coccoid form, the majority of the ∆*pgp1* mutant population was rod‐shaped albeit with a larger amount of coccoid cells than at 5 h (Fig. 6B). Even at 48 h there were still a few straight bacteria in the intracellular ∆*pgp1* mutant population (Fig. 6C).

### Intestinal epithelial cells are unresponsive to coccoid *C. jejuni* cells

#### Coccoid peptidoglycan causes a drastic decrease in Nod1 and Nod2 signaling in comparison to wild type

**Figure 7:**
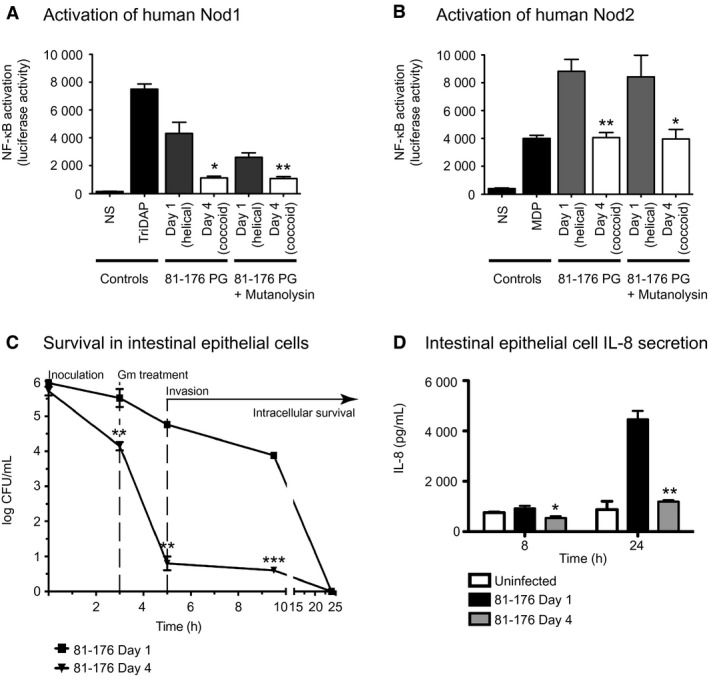
Intestinal epithelial cells were unresponsive to coccoid *C. jejuni* cells: coccoid *C. jejuni* PG caused a decrease in human Nod1 and Nod2 signaling and coccoid cultures showed minimal invasion and intracellular survival as well as IL‐8 production. A‐B, to compare the ability of helical and coccoid *C. jejuni* PG to activate Nod proteins, human embryonic kidney cells (HEK293T) were co‐transfected with either 0.1 µg/mL whole PG or muropeptides (from mutanolysin digested PG) of the *C. jejuni* wild‐type strain 81‐176 grown on plates for 1 day (helical) or 4 days (coccoid) *C. jejuni* 81‐176, vectors for a nuclear factor‐κB (NF‐κB) luciferase reporter, and either human Nod1 (A) or Nod2 (B). Nod activation was determined by measuring the activity of a NF‐κB luciferase reporter in comparison to the non‐stimulated (NS) negative control. Positive controls used were TriDAP and MDP. C, a gentamicin (Gm) protection assay was used to assess the invasion and intracellular survival in INT407 epithelial cells of a mainly coccoid culture of *C. jejuni* 81‐176 grown for 4 days in broth in comparison to a helical population grown for 1 day. Gm was added 3 h post‐infection to remove all extracellular bacteria, and after 2 h the Gm was washed off and the cells were incubated with fresh MEM containing 3% FBS and a low dose of Gm. At each timepoint, cells were lysed with water and CFUs were determined for each well by plating on MH‐TV plates. D, IL‐8 levels secreted by uninfected INT407 epithelial cell lines and cells infected for 8 and 24 h with *C. jejuni* wild type 81‐176 grown on plates for one day (helical) or four days (coccoid) were quantified by ELISA. In all experiments standard errors of the mean were calculated from triplicate readings and are representative of three independent experiments. The asterisk (*) indicates a statistically significant difference using the unpaired Student's t‐test between the 81‐176 Day 1 and Day 4 samples, with *, ** or *** indicating *p* < 0.05, *p* < 0.01, and *p* < 0.0001, respectively. There was no significant difference between the IL‐8 levels in the uninfected and 81‐176 Day 4 samples in D.

To determine whether Nod receptor stimulation was altered as a result of PG changes in a coccoid population, expression of an NF‐κB *lgk* luciferase reporter was transfected in human embryonic kidney HEK293T cells along with either the human Nod1 (Fig. 7A) or Nod2 (Fig. 7B) receptor and whole PG or muropeptides (from mutanolysin digested PG). PG was isolated from either *C. jejuni* wild‐type grown on solid media for 1 day (helical) or 4 days (coccoid) at 38°C. Day 4 coccoid PG and muropeptides, respectively, showed a 3.9‐ and 2.5‐fold statistically significant reduction in Nod1 stimulation in comparison to Day 1 helical samples. This was in accordance with a decrease in the tripeptide Nod1 agonist in coccoid PG. For Nod 2, Day 4 coccoid PG and muropeptides showed the same level of activation as the muramyldipeptide (MDP) positive control, but a respective 2.2‐ and 2.1‐fold lower level of stimulation in comparison to Day 1 helical samples, despite an increase in the dipeptide Nod2 agonist in the coccoid PG. The use of undigested polymeric PG versus monomeric PG generated by mutanolysin did not result in a statistically different change in activation levels for neither Day 1 or Day 4 samples, nor Nod1 or Nod2 receptors.

#### Coccoid bacteria exhibit a defect in adherence, invasion and intracellular survival in epithelial cells

A Gm protection assay was used to assess the adherence, invasion, and intracellular survival properties of a *C. jejuni* coccoid culture (Fig. 7C). The coccoid (81‐176 Day 4) population showed a 1.4‐, 4.0‐, and 3.3 log‐fold reduction in recovery from INT407 intestinal epithelial cells in comparison to the helical (81‐176 Day 1) population at the 3 h adherence and invasion, 5 h invasion, and 9.5 intracellular survival timepoints, respectively. By 24 h post‐infection, bacteria from neither the helical nor the coccoid samples were recovered.

At each timepoint, CFUs were also determined by plating on MH‐TV plates with and without 20 mM sulfite (data not shown). The addition of sulfite has been shown to enhance *C. jejuni* recovery post‐host cell infection (Pryjma *et al.*, 2012). In this experiment, the addition of sulfite to the helical (81‐176 Day 1) and to the coccoid (81‐176 Day 4) samples resulted only in a minor improvement in recovery (data not shown).

#### Coccoid cells do not induce IL‐8 secretion from epithelial cells

Epithelial cell infections with *C. jejuni* produce an IL‐8 response (van Putten *et al.*, 2009). The levels of IL‐8 in the supernatant of INT407 human epithelial cell lines exposed to an equal number of CFU/ml of helical (81‐176 Day 1) and coccoid (81‐176 Day 4) populations of *C. jejuni* were measured by ELISA (Fig. 7D). There was no statistically significant difference in the IL‐8 levels of the uninfected cells and those exposed to the coccoid populations. The helical *C. jejuni* population produced a statistically significant 1.7‐ and 3.7‐fold higher IL‐8 response at 8 h and 24 h, respectively, in comparison to the coccoid population.

## Discussion

*C. jejuni* entrance into the VBNC state and transition to the coccoid form are distinct but related events in the lifecycle of this organism (Ikeda and Karlyshev, 2012). This study focused solely on examining characteristics of the coccoid form and its formation: the muropeptide profile of coccoid PG, PG hydrolases involved in the transition to the coccoid form, and the response of intestinal epithelial cells to coccoid *C. jejuni*.

### Coccoid *C. jejuni*

Environmental conditions [such as starvation, suboptimal temperatures, changes in oxygen tension, pH, osmolarity, and pressure (reviewed in Svensson *et al.*, 2008; Ikeda and Karlyshev, 2012)] and now, intracellular survival (Fig. 6), result in *C. jejuni* transitioning from a helical to a coccoid form. As expected, this change in morphology was accompanied by remodeling of the PG. Similar to coccoid *H. pylori* (Costa *et al.*, 1999), the muropeptide profile of coccoid *C. jejuni* was characterized by increased amounts of dipeptides and reduced total tripeptides and monomeric tetrapeptides. These changes were consistent at both conditions used to examine coccoid formation in *C. jejuni*: (1) starvation (prolonged incubation at 38°C), and (2) temperature and oxidative stress (incubation at 4°C under aerobic conditions). A few additional changes occurred under condition (2) that may be a result of the environmental changes or differences in growth phase. Coccoid *H. pylori* muropeptides also showed increases in cross‐linking and anhydromuropeptides not present in coccoid *C. jejuni*; however, these changes appear to result from differences in growth phase (Costa *et al.*, 1999; Chaput *et al.*, 2006), as has been seen for *E. coli* (Pisabarro *et al.*, 1985).

*C. jejuni* can survive and maintain its metabolic activities at 4°C for long periods of time (Hazeleger *et al.*, 1998), although it cannot grow at this temperature or in aerobic environments. Since *C. jejuni* becomes coccoid under non‐permissive growth conditions, this suggests that growth is not required for coccoid formation. This is supported by the results of Hazeleger *et al.* (1995) that showed there was no alteration in protein profile as cells transitioned to a coccoid form and that coccoid cells still formed at the same rate when protein synthesis was blocked and when DNA was damaged by irradiation. However, our study did show an increase in *pgp1* transcript levels during the time period over which *C. jejuni* transitioned to coccoid.

### Pathogenesis of the coccoid form

The *C. jejuni* coccoid population has been suggested by some to be a degenerate form of the organism (Ikeda and Karlyshev, 2012). However, as has been noted for *H. pylori* (Chaput *et al.*, 2006), since PG remodeling is involved in the transition to a coccoid form in both *C. jejuni* and *H. pylori*, one would expect this process to be regulated and serve a biological purpose rather than result in a degenerate form. Changes in morphology and muropeptide structure will also affect the pathogenic properties of the organism and how it is recognized by the host immune system (Frirdich *et al.*, 2012; 2014; Stahl *et al.*, 2016). The effect of coccoid cells on host‐pathogen interactions was determined by examining the ability of coccoid cells to adhere and invade epithelial cells, the response of epithelial cells to the presence of coccoid cells (through IL‐8 production, an early signaling molecule in the mucosal inflammatory response), and the activation of Nod1 and Nod2 epithelial cell receptors by coccoid PG. In all instances, epithelial cells were found to be completely unresponsive to coccoid cells.

The adherence and invasion of helical and coccoid *C. jejuni* cells in epithelial ceIls was examined using a Gm protection assay using an equivalent CFU/mL of 81‐76 (98.4% helical) Day 1 and Day 4 (97.8% coccoid) cultures at the start of the infection process. The amount of cells adhered and invaded after a 3 h infection period were approximately 46.2% of the starting inoclulum for the Day 1 helical cultures and only 2.6% of the Day 4 cultures. A 3 day culture of *C. jejuni* M129 that was > 70% coccoid also showed a significant decrease in adherence to INT407 cells (Konkel *et al.*, 1992). The low level of adherence and invasion by Day 4 coccoid cultures could potentially be due to the remaining helical cells in the population (which make up approximately 1.4 ± 0.7% of the population) if all the helical cells adhered to the intestinal cells. Attempts to image the GFP‐tagged Day 4 *C. jejuni* cells adhering and invading to intestinal epithelial cells to determine whether only the remaining helical cells adhered and invaded were unsuccessful. Several factors such as differences in growth phase, morphology, and motility are likely contributing to the lack of adherence and invasion of INT407 cells by the coccoid cultures. All factors are interdependent and vary as the bacteria age.

The presence of wild‐type flagella and motility are essential to *C. jejuni* adherence and invasion (Backert and Hofreuter, 2013; Mertins *et al.*, 2013). Coccoid *C. jejuni* were non‐motile despite the presence of flagella (Fig. S3), as has been reported previously (Moran and Upton, 1987; Ikeda and Karlyshev, 2012). *C. jejuni* mutants in *motAB* and *pflA* form wild‐type flagella, but are incapable of flagellar rotation and are non‐motile (Zheng *et al.*, 2008; Mertins *et al.*, 2013). These mutants adhere to epithelial cells, but show a significant reduction in invasion. Full‐length flagella, but not motility, are required for adherence, supporting the idea that flagella may act as an adhesin (Yao *et al.*, 1994). In our study, despite the presence of flagella (Fig. S3), only a very small fraction of the *C. jejuni* 81‐176 Day 4 coccoid population adhered to intestinal epithelial cells. This could be due to a number of factors such as: a reduction in flagellar number, changes in flagellar structure not detected microscopically, alteration of another adherence factor, or differences in stress survival characteristics preventing survival of coccoid *C. jejuni* in the presence of epithelial cells.

Isolated PG and muropeptides of *C. jejuni* helical and coccoid cells differentially activated cytoplasmic Nod receptors, with coccoid PG decreasing activation levels of both Nod1 and Nod2. As expected, decreased tripeptide Nod1 agonist in coccoid samples resulted in a corresponding decrease in Nod1 stimulation. Unexpectedly, Nod2 activation also decreased with coccoid samples, despite a significant increase in the canonical dipeptide Nod2 agonist in the coccoid PG. As only PG and not whole cells were used in this assay, only the response to PG was measured. Therefore, the lack of adherence and invasion of coccoid *C. jejuni* would have no bearing on Nod activation levels, and it is not possible for factors other than PG structural changes to interfere with the assay. An as‐of‐yet unidentified modification present on the coccoid PG may be interfering with recognition of the coccoid dipeptides by Nod2. The presence of anhydropeptides has been shown to affect signaling through Nod2 (Chaput *et al.*, 2006). However, no anhydrodipeptides were detected in the coccoid *C. jejuni* muropeptide profile. Nod2 signaling in response to muropeptides from other *C. jejuni* strains also gave unexpected results: despite a decrease in dipeptides in ∆*pgp1* and ∆*pgp2* PG, there was no change in Nod2 activation levels in comparison to wild type (Frirdich *et al.*, 2012; 2014). Nod2 is a cytosolic receptor protein that associates with the membrane. This membrane association is critical for activating the downstream NF‐κB response (Barnich *et al.*, 2005; Lipinski *et al.*, 2012; Philpott *et al.*, 2014). Membrane proteins and/or Nod2 interaction partners may be affecting Nod2 ligand specificity and activation in response to *C. jejuni* coccoid muropeptides, as proteins have been shown to interact with Nod2 and enhance or inhibit signaling through NF‐κB (Boyle *et al.*, 2014). A further general understanding of Nod2 activation is required in order to develop a hypothesis as to why Nod2 did not respond to coccoid *C. jejuni* muropeptides.

Similar to other enteric pathogens*, C. jejuni* activates the NF‐κB transcription factor in epithelial cells, triggering a pro‐inflammatory response and the secretion of IL‐8 (Hickey *et al.*, 1999; 2000; Mellits *et al.*, 2002; MacCallum *et al.*, 2006). IL‐8 production in response to *C. jejuni* has been shown to occur by different pathways. These include bacterial attachment and invasion (Hickey *et al.*, 1999), bacterial protein synthesis stimulated by contact with epithelial cells (Watson and Galan, 2005), and by the *C. jejuni* cytolethal distending toxin (CDT) (Hickey *et al.*, 2000). This suggests that IL‐8 secretion occurs via the recognition of several different cellular components, although the specific *C. jejuni* factors involved are currently unknown. Host sensing of bacterial components to activate NF‐κB occurs through Toll‐like receptors (TLRs) that sense conserved microbial structures and Nod proteins (Nod1 and Nod2) sensing intracellular bacterial PG. One study showed that *C. jejuni* triggering of IL‐8 secretion was dependent on TLR activation, likely mainly through TLR2 recognizing lipoproteins and/or TLR4 recognizing lipopolysaccharides (Zheng *et al.*, 2008). However, *C. jejuni* was also reported to activate NF‐κB independently of TLR and Nod proteins, with TLR2 and Nod proteins enhancing NF‐κB activation (Al‐Sayeqh *et al.*, 2010).

Coccoid *C. jejuni* did not induce IL‐8 secretion from INT407 intestinal epithelial cells. Considering what is known regarding *C. jejuni* IL‐8 secretion, several factors could explain this observation: the inability of coccoid cells to adhere to and invade epithelial cells, a lack of protein synthesis in response to epithelial cells as coccoid formation was accompanied by a decrease in protein synthesis (Ikeda and Karlyshev, 2012), the potential lack of CDT production (it is unknown whether CDT is produced by coccoid cells), a change in surface components that interact with epithelial cells, and PG that does not stimulate Nod1 and Nod2.

Coccoid *H. pylori* cells were also unable to trigger an immune response. As with *C. jejuni*, the *H. pylori* transition to coccoid is accompanied by a decrease in muropeptide tripeptides and a corresponding decrease in Nod1 activation by isolated coccoid PG muropeptides (Chaput *et al.*, 2006). Unlike helical *H. pylori*, coccoid cells did not activate NF‐κB or release of IL‐8 from AGS gastric epithelial cells (Chaput *et al.*, 2006). The lack of immune response to coccoid *H. pylori* could potentially be a form of immune escape that results in chronic infection (Chaput *et al.*, 2006). This may also be the case for *C. jejuni*.

### PG hydrolases involved in coccoid formation

Since Pgp1 is a PG hydrolase with DL‐carboxypeptidase activity cleaving tri‐ to dipeptides, and coccoid *C. jejuni* has increased dipeptides and decreased tripeptides, the involvement of Pgp1 in coccoid formation was expected. A ∆*pgp1* mutant was delayed in coccoid formation during prolonged incubation in media and within epithelial cells. However, the ∆*pgp1* mutant population still became mainly coccoid over time, indicating that additional PG hydrolases to Pgp1 are involved in remodeling the PG during the transition to the coccoid form. The amidase AmiA was also found to play a role in the transition, with an ∆*amiA* mutant exhibiting partial coccoid formation after 8 days of growth on solid media, and a ∆*amiA*∆*pgp1* double mutant being largely unable to transition a coccoid form, equivalent to a single ∆*amiA* mutant in *H. pylori* (Chaput *et al.*, 2006). Other mutants identified that produced cells of increased cell length or chains of cells similar to the morphology of Δ*amiA*  still retained the ability to transition to the coccoid form (data not shown).

Unlike *H. pylori* ∆*amiA* that was defective for dipeptide accumulation and predominantly non‐coccoid, the ability to accumulate dipeptides was unaffected in the partially coccoid *C. jejuni* ∆*amiA* mutant population. The general trend in muropeptide profile changes over time of the non‐coccoid ∆*amiA*∆*pgp1* double mutant were more similar to that of *H. pylori* ∆*amiA* (Chaput *et al.*, 2016). The role of the *H. pylori* Pgp1 homolog Csd4 in coccoid formation in that organism remains to be determined. The role of the *H. pylori* Pgp1 homolog Csd4 in coccoid formation remains to be determined, although the inability of a *H. pylori amiA* mutant to accumulate dipeptides would suggest that Csd4 may be impaired in this mutant. As suggested by Chaput *et al.* (2016), if Csd4 and AmiA are found in a protein complex, loss of AmiA and protein‐protein interactions with Csd4 could disrupt the complex affecting Csd4 activity.

### *C. jejuni* AmiA

The *C. jejuni* amidase was previously uncharacterized. Genomic analyses indicated that *C. jejuni*, as with the related *H. pylori*, encodes a single N‐acetylmuramoyl‐L‐alanine amidase annotated AmiA. Similar to *H. pylori*, *C. jejuni* AmiA played an important role in septum cleavage during cell division, with a mutant strain producing long chains of unseparated cells. This phenotype is similar to that of an *E. coli* mutant lacking all three periplasmic amidases, AmiA, AmiB and AmiC (Heidrich *et al.*, 2001; 2002). The *E. coli ∆amiA∆amiB∆amiB* mutant displayed a growth defect, but not to the same extent as that of *C. jejuni* ∆*amiA*. Passage of the *C. jejuni* ∆*amiA* strain improved the culturability and cell separation defects. Suppressor mutations were not identified in these passaged mutants. Therefore, it is possible that protein expression differences account for the suppressor phenotype.

The effect of an *amiA* deletion in *C. jejuni* differed slightly than that of *H. pylori*. The growth of *C. jejuni ∆amiA* was compromised, unlike *H. pylori* (Chaput *et al.*, 2006). Both are non‐motile; however, *C. jejuni* ∆*amiA* did not form flagella while *H. pylori ∆amiA* still assembles flagella at the poles and some division sites, but these flagella were paralyzed (Chaput *et al.*, 2016). It remains to be determined how the loss of *amiA* affects flagellar assembly in *C. jejuni* and flagellar function in *H. pylori*. One explanation may be that in *C. jejuni* amidase activity is necessary for the insertion of the flagellar secretion apparatus through the PG layer. A PG hydrolase involved in this process has yet to be identified in *C. jejuni*. Another hypothesis is that amidase activity and cell separation may be a signal for flagellar assembly.

N‐acetylmuramoyl‐L‐alanine amidases cleave the PG peptide from the glycan strand. The muropeptide profile of a *C. jejuni* ∆*amiA* mutant after one day of growth was unchanged, suggesting that AmiA has no substrate preference in terms of peptide length or degree of cross‐linkage. Overexpression of *C. jejuni* AmiA did show a slight increase in dipeptides. This may indicate a subtle preference for peptide sidechains longer than dipeptides. Alternatively, the increased level of dipeptides may reflect a concomitant increase in hydrolase activity such as that of Pgp1, which generates dipeptides. Enzyme activity assays will be required to determine the precise substrate specificity of *C. jejuni* AmiA. Amidase mutants from various organisms display different muropeptide profiles. The *V. cholerae* ∆*amiB* mutant has increased dipeptides, indicating that AmiB is required for dipeptide release (Moll *et al.*, 2014), while the *H. pylori* ∆*amiA* mutant has decreased dipeptides and shows a defect in dipeptide accumulation over time. The *H. pylori* ∆*amiA* mutant also appears to have longer glycan chain lengths, while in *E. coli* ∆*amiA*∆*amiB*∆*amiC*, the muropeptide profile shows shorter chain lengths, a decrease in monomers and elevated levels of trimers and tetramers (Heidrich *et al.*, 2001).

Amidase protein sequences and the biological effect of amidase loss, including changes to the muropeptide profile differ significantly between organisms, even closely related organisms such as *C. jejuni* and *H. pylori*. This emphasizes the importance of studying cell division and PG biosynthesis in different organisms rather than assuming that each amidase homolog affects these processes in a similar manner.

#### Strains defective in coccoid formation still accumulate muropeptide dipeptides, but show minimal changes in monomeric tripeptides

Examination of the muropeptide profile of strains that by Day 4 were nearly completely coccoid (81‐176, ∆*pgp2* and 81‐176 overexpressing *pgp1*), partially coccoid (∆*pgp1* and ∆*amiA*), or did not go coccoid (∆*pgp1*∆*amiA*) showed that coccoid cultures had lower levels of monomeric tripeptides and higher levels of tetrapeptides than the other strains (Table S5). There was no distinguishing characteristic of the completely non‐coccoid muropeptide profile in comparison to the partially coccoid strains. Comparing the changes that occurred from Day 1 to Day 4, all strains regardless of their morphology, showed an increase in dipeptides and a decrease in tetrapeptides (Table S5). What seems to distinguish the non‐coccoid and partially coccoid forms from the coccoid strains is a lack of change in tripeptides, with the exception of ∆*pgp2*. The muropeptide profile of ∆*pgp2* has no tripeptides due to the loss of the LD‐carboxypeptidase activity of Pgp2 cleaving tetra‐ to tripeptides (Frirdich *et al.*, 2014), but still transitioned to a coccoid form and even accumulated small amounts of tripeptides by Day 4. In *H. pylori*, the ∆*amiA* mutant defective for coccoid formation also did not show significant changes in tripeptides [Table S6, (Chaput *et al.*, 2016)]. Examination of coccoid formation in additional *C. jejuni* mutants will help confirm this hypothesis. The *∆amiA* and *∆amiA∆pgp1* strains also showed a larger change in monomeric tetrapeptides. This may be a side effect of *∆amiA* deletion or, as these were the least coccoid strains examined, may also play a role in preventing coccoid formation.

A mutant in *pgp1*, despite being unable to cleave tripeptides to dipeptides, still accumulated dipeptides as it aged. As all strains accumulated dipeptides and showed a reduction in tetrapeptides, it is possible that the dipeptides are generated by cleavage of the tetrapeptides by a yet unidentified DL‐endopeptidase. This is supported by the muropeptide profile of a *∆pgp2* mutant that also shows the presence of dipeptides which have to be generated by a hydrolase other than Pgp1, as *∆pgp2* completely lacks the tripeptide substrate for Pgp1. Despite the absence of Pgp1 substrate in *∆pgp2* and Pgp1 itself in *∆pgp1∆pgp2*, these strains show no delay in coccoid formation. In these strains, the ∆*pgp2* mutation is suppressing the defect in coccoid transition arising from a lack of Pgp1 activity. One possibility is that the loss of Pgp2 is increasing the activity of another hydrolase involved in coccoid formation. A better understanding of the *C. jejuni* PG biosynthetic complexes and protein‐protein interactions with Pgp1 and Pgp2 is required to interpret these results.

### Future considerations

This study characterized the PG muropeptide profile of coccoid *C. jejuni* strains and identified Pgp1 and AmiA as factors involved in the shift from a helical to coccoid morphology. Additional proteins are likely to be involved as well. If indeed the coccoid form is important in the lifecycle of *C. jejuni*, reversion back to the helical form would be expected. There have been some controversial reports of *C. jejuni* coccoid‐to‐helical reversion (Ikeda and Karlyshev, 2012). If *C. jejuni* is coccoid under environmental conditions, it is expected to return to a motile helical morphotype to infect the human gastrointestinal tract and invade intestinal epithelial cells. Once within epithelial cells, we have shown that over time *C. jejuni* transitions to a coccoid form. Coccoid *C. jejuni* do not activate the intracellular Nod1 and Nod2 receptors of the innate immune system or trigger IL‐8 secretion. These coccoid bacteria may serve as a means of escaping the immune system and as a reservoir for the development of chronic *C. jejuni* infections (Nachamkin, 2002; Sherman *et al.*, 2010; Riddle *et al.*, 2012; Bolton, 2015). As well, the presence of intracellular coccoid bacteria that are immunologically invisible may explain the recrudescence observed in the experimental *C. jejuni* infection of human volunteers (Baqar *et al.*, 2010; Kirkpatrick and Tribble, 2011; Rimmer *et al.*, 2018).

The closely related helical organisms *C. jejuni* and *H. pylori* both have the capacity to evolve into coccoid cells under prolonged incubation; however, despite the existence of similarities in the PG biosynthetic pathways of both organisms, interesting differences exist, such as the different roles of Pgp1 and AmiA in the coccoid transformation process. This highlights the importance of studying PG biosynthetic pathways and mechanisms underlying morphological changes even in related organisms.

## Experimental procedures

### Bacterial strains and growth conditions

The bacterial strains and plasmids used in this study and their construction are described in the Supplemental Experimental Procedures. Unless otherwise indicated, *C. jejuni* strains were grown at 38°C in Mueller‐Hinton (MH; Oxoid) broth or on 8.5% (w/v) agar plates supplemented with vancomycin (10 μg/ml) and trimethoprim (5 μg/ml), denoted MH‐TV, under microaerobic/capnophilic conditions (6% O_2_, 12% CO_2_; hereafter designated as microaerobic) in a Sanyo tri‐gas incubator for plates or using the Oxoid CampyGen system for broth cultures. Growth media was supplemented with chloramphenicol (20 μg/ml) or kanamycin (50 μg/ml), where appropriate. *E. coli* strains used for plasmid construction were grown at 38°C in Luria‐Bertani (LB; Sigma) broth or 7.5% agar (w/v) agar plates and supplemented with ampicillin (100 μg/ml), chloramphenicol (15 μg/ml), or kanamycin (25 μg/ml), as necessary.

For growth analyses, *C. jejuni* strains were streaked from 16 h plate cultures and grown again on plates for 5–7 h. Bacteria were harvested in MH‐TV broth and inoculated at an OD_600_ of 0.002 into MH‐TV broth and grown shaking for 18 h. Strains were subcultured to an OD_600_ of 0.002 and samples were taken at various timepoints for colony forming unit (CFU) and microscopic analysis.

### Microscopy

For visualization under bright field or DIC microscopy, 1 μl of a broth or plate culture was immobilized on a thin 1% agar (w/v in H_2_0) slab and overlayed with a cover slip. Images were captured with a Nikon Eclipse TE2000‐U microscope equipped with 100x objective and a Hamamatsu Orca camera system. Quantification of the percentage of helical, coccoid and cells transitioning to the coccoid form in the DIC images was carried out by counting the number of each using ImageJ software (NIH). At least three separate fields of view of approximately 200 bacteria were counted for each strain at each timepoint and this was carried out for three separate cultures.

TEM was carried out on shaking MH‐TV broth or plate cultures. Samples were fixed in a final concentration of 2.5% (v/v) of gluteraldehyde for 2‐3 h on ice. Cells were harvested, resuspended in an equal volume of H_2_O, and stored at 4°C. For imaging, 2 μL of bacteria was spotted onto parafilm to which 4 μL of 0.5% uranyl acetate was added for 1 min. A formvar‐carbon film on 300 mesh copper grid (Canemco, Lakefield, Quebec, Canada) was added to the bacteria‐uranyl acetate spot for 2 min. The grid was then removed, dried, washed ten times in sterile water, dried again and visualized on a Hitachi H7600 TEM equipped with a side mount AMT Advantage (1 mega‐pixel) CCD camera (Hamamatsu ORCA) at the UBC Bioimaging facility (The University of British Columbia, Vancouver, BC, Canada).

### Real‐time quantitative PCR (RT‐qPCR)

*C. jejuni* 81‐176 was grown under microaerophilic conditions at 38°C on solid media. At each timepoint, RNA was isolated and cDNA was synthesized as described (Svensson *et al.*, 2009). Quantitative PCR was performed using primers designed to *pgp1* and *rpoA*, used as an internal control (see Supplemental Experimental Procedures for primer sequences). Reactions were set up with IQ SYBR green Supermix (Bio‐Rad) and performed with a CFX96 real‐time PCR detection system (Bio‐Rad). Expression differences were calculated by using the ΔΔ*C_T_* method. Data are representative of three independent experiments.

### Motility

Motility assays were carried out with strains grown either for 18 h in shaking MH‐TV broth or on plates. Cultures were standardized to an OD_600_ of 0.2 in MH‐TV and 2 μl were point inoculated into MH‐TV plates containing 0.4% agar. Plates were incubated for 20 h and the halo diameter was measured.

### Peptidoglycan isolation and muropeptide analysis

*C. jejuni* strains were passaged once from frozen stocks and then passaged to 20–25 MH plates and grown for either 18–20 h (day 1 samples) or 76 h (day 4 samples) at a final OD of 200–600. Cells were collected into cold MH broth by scraping, harvested by centrifugation at 8000 × g for 15 min and then resuspended in 6 ml ice cold H_2_O. Cells were lysed by dropwise addition to 6 mL 8% SDS boiling under reflux. PG was purified from the cell lysate (day 4 samples required extra washing steps to remove the SDS), digested with the muramidase cellosyl (kindly provided by Hoechst, Frankfurt, Germany), and the resulting muropeptides were reduced with sodium borohydride and separated by HPLC as described (Glauner, 1988). Muropeptide structures were assigned based on (i) comparison with retention times of known muropeptides from *C. jejuni* (Frirdich *et al.*, 2012) and (ii) by mass spectrometry (MS). For MS analysis, muropeptide fractions were collected, concentrated in a SpeedVac, acidified by 1% trifluoroacetic acid, and analysed by offline electrospray mass spectrometry on a Finnigan LTQ‐FT mass spectrometer (ThermoElectron, Bremen, Germany) at the Newcastle University Pinnacle facility as described (Bui *et al.*, 2009).

### *In vitro* invasion and intracellular survival in epithelial cell lines and confocal microscopy of internalized cells

The human epithelial INT407 cell line was used for *C. jejuni* infections. Media used for growth of the cell lines was as directed by the ATCC. Cells were seeded into 24‐well tissue culture plates at semiconfluence at approximately 1 × 10^5^ cells/ml and allowed to grow for 20–24 h prior to infection. Infections were carried out as described (Frirdich *et al.*, 2012), with approximately 1 × 10^7^ CFU/ml of either the *C. jejuni* wild‐type strain 81‐176 grown in shaking broth culture for one day (81‐176 Day 1, helical morphology) or four days (81‐176 Day 4, coccoid morphology). An OD_600_ of 0.002 and 0.15 was equivalent to 1 × 10^7^ CFU/ml for *C. jejuni* grown for one day and four days, respectively. Standard errors of the mean were calculated from triplicate readings and are representative of three independent experiments.

To visualize *C. jejuni* cells that had been internalized by epithelial cells using confocal microscopy, strains were transformed with the pRY112‐*P_atpF'_‐gfp* plasmid (Apel *et al.*, 2012) expressing GFP from the *C. jejuni atpF’* promoter. Confocal microscopy was performed as described (Pryjma *et al.*, 2012). Briefly, INT407 epithelial cells were grown to semiconfluence and seeded onto glass coverslips (Fisher) at 1.5 × 10^5^ cells per well and grown for 20–24 h. Shaking broth cultures of *C. jejuni* wild‐type 81‐176 or *Δpgp1* inoculated at an OD_600_ of 0.002 were grown for 18 h. Bacteria were washed 2 times with MEM and used to infect the INT407 monolayers at 1 × 10^6^ bacterial cells/ml in MEM, an MOI of 10. Infection and Gm treatment were performed as described (Pryjma *et al.*, 2012). At each time point, monolayers were washed twice with phosphate‐buffered saline (PBS) before fixation with 4% paraformaldehyde (Canemco) in PBS. Samples were mounted using Prolong Gold Antifade with DAPI (Invitrogen). Imaging was performed with an Olympus Fluoview FV1000 laser scanning confocal microscope using FV10‐ASW 2.0 Viewer software to adjust the images. Three fields of view were visualized for each sample. Each experiment was performed in triplicate on duplicate samples of each strain.

### Epithelial cell responses & Nod activation assays

Luciferase asssays were performed as previously described (Lee *et al.*, 2009; Frirdich *et al.*, 2012). Briefly, HEK293T cells were transfected overnight with 75 ng of NF‐κB luciferase reporter plasmid (Igκ‐luc, Invitrogen) and either human Nod1 (3 ng) or Nod2 (0.1 ng). The empty vector (pcDNA3.1, Invitrogen) was used to balance the transfected DNA concentration. At the same time, 0.1 μg/ml of either whole PG or muropeptides of the *C. jejuni* wild‐type strain 81‐176 grown on plates for one day (helical) or four days (coccoid) were added, and the NFκB‐dependent luciferase activation was then measured following 18–24 h of co‐incubation. Positive controls were TriDAP (tripeptide L‐Ala‐γ‐D‐Glu‐*meso*‐DAP, 5 μg/ml) and MDP (10 μg/ml) for Nod1 and Nod2 assays, respectively. Four independent experiments were carried out each with triplicate samples. The data shown represents one experiment, but similar findings were seen in all four trials.

### Interleukin‐8 quantification

The INT407 human epithelial cell line was seeded into 24‐well tissue culture plates at approximately 1 × 10^5^ cells/ml in MEM supplemented with 10% FBS and allowed to grow for 20–24 h prior to infection. The cells were washed three times with MEM and either left uninfected or infected with approximately 1 × 10^7^ cells/ml of either the *C. jejuni* wild‐type strain 81‐176 grown in shaking broth culture for one day or four days. Supernatants were collected after 8 h and 24 h of co‐infection, centrifuged for 10 min to pellet residual cells and bacteria, and frozen at −80°C until assayed. The concentration of IL‐8 present in the supernatants was measured by the human IL‐8 ELISA kit (Invitrogen, Camarillo, CA). Each experiment was carried out with triplicate samples and was repeated to give a total of three experiments. Data shown are representative of all three experiments.

## Author contributions

Conceived and designed the experiments: EF, JB, JL, SH, CTP, SEG, WV; Performed the experiments: EF, JB, MP, JL, SH; Analyzed and interpreted the data: EF, JB, MP, JL, SH, CTP, SEG, WV, ECG; Drafting and critically reviewing the manuscript: EF, JB, CTP, SEG, WV, ECG.
