# Supplementary material for: The Campylobacter jejuni helical to coccoid transition involves changes to peptidoglycan and the ability to elicit an immune response
Source: Mol Microbiol. 2019 May 20;112(1):280–301. doi: 10.1111/mmi.14269 (PMC6767375; doi:10.1111/mmi.14269)
Supplement: Supplementary file 1 [file MMI-112-280-s001.pdf]

Supplemental information

The *Campylobacter jejuni* helical to coccoid transition involves changes to peptidoglycan and the ability to elicit an immune response

Emilisa Frirdich[1*], Jacob Biboy[2], Mark Pryjma[2], Jooeun Lee[3], Steven Huynh[4], Craig T. Parker[4], Stephen E. Girardin[3], Waldemar Vollmer[2], and Erin C. Gaynor[1*]

Running title: *C. jejuni* helical to coccoid transition

[1] Department of Microbiology and Immunology, University of British Columbia, Vancouver, BC, Canada
[2] The Centre for Bacterial Cell Biology, Institute for Cell and Molecular Biosciences, Newcastle University, Newcastle upon Tyne, United Kingdom
[3]Department of Laboratory Medicine and Pathobiology, University of Toronto, Toronto, ON, Canada
[4]Produce Safety and Microbiology Unit, Western Region Research Center, USDA, Agricultural Research Service, Albany, CA, USA

* Address correspondence to:
Emilisa Frirdich: emilisa@mail.ubc.ca, 604-827-5796;
or Erin C. Gaynor: egaynor@mail.ubc.ca, 604-822-6042

Key words: *Campylobacter jejuni*, cell morphology, peptidoglycan, DL carboxypeptidase, amidase

List of Contents

Supplemental Experimental Procedures

Strain construction (including table of bacterial strains and plasmids and table of primers used in this study)

Whole genome sequencing

Supplemental Tables:

Table S1

Table S2

Table S3

Table S4A
Table S4B

Table S5

Table S6

Supplemental Figures:

Figure S1. The *C. jejuni* transition of Δ*pgp2* and Δ*pgp1*Δ*pgp2* mutant strains from a helical to coccoid form was similar to wild type.

Figure S2. Amidase multiple sequence alignment.

Figure S3. Coccoid *C. jejuni* retained their flagella.

References

Supplemental experimental procedures

Construction of *C. jejuni* 81-176 Δ*amiA*, Δ*1284*, the complemented Δ*amiA* strain, and Δ*pgp1* Δ*amiA*.

In order to delete the *amiA* gene, a portion of the gene was replaced by the Cm resistance cassette (*cat*). Initial attempts to replace a portion of the *C. jejuni amiA* gene with the non-polar *aphA3* kanamycin (Km) resistance cassette from pUC18-Km (Menard *et al.*, 1993) were unsuccessful. The *amiA* gene was PCR amplified with iProof (Biorad) from *C. jejuni* 81-176 genomic DNA using primers amiA-1 and amiA-2 (3060 bp). A polyA tag was added to the PCR product and it was ligated to a commercially available pGEM-T vector (Promega). The resulting construct pEF18 was verified by PCR analysis and sequencing. Inverse PCR was performed on pEF18 with primers amiA-3 and amiA-5 (deleting nucleotides 127-1664 of *amiA*) and the product was ligated to a *cat* cassette digested out of pRY109 (Yao *et al.*, 1993) with *SmaI* to form plasmid pEF18Cm. Correct insertion of the cassette in the same orientation as the *amiA* gene was verified by PCR with cat-2 and amiA-2 primers, as well as restriction enzyme analysis. *C. jejuni* 81-176 was naturally transformed with pEF18Cm and mutants were selected on MH-TV containing Cm. Mutants (designated *ΔamiA*) were very slow growing and appeared after 5-7 days of growth as very small colonies. They were verified by PCR analysis with amiA-6 and amiA-7.

To verify that phenotypes seen with Δ*amiA* were not due to polar effects on the downstream gene 1284 (CJJ81176_1284; *mnmC*), the downstream gene was deleted. Gene 1284 was PCR amplified from 81-176 genomic DNA with primers 1284-1 and 1284-2 (2711 bp), a polyA tail was added to the PCR product and it was cloned into pGEM-T, forming construct pEF74. The pEF74 plasmid was verified by PCR analysis and sequencing. Inverse PCR was performed on pEF74 with primers 1284-3 and 1284-4 to delete nucleotides 37-1692 of the 1842 bp gene. The inverse PCR product was digested with *DpnI* (to remove any native pEF74) and then ligated to the *SmaI*-digested *cat* cassette from pRY109 (Yao *et al.*, 1993), resulting in pEF74Cm. Correct insertion of the cassette in the same orientation as the *1284* gene was verified by PCR with cat-2 and 1284-2 primers, as well as restriction enzyme analysis. *C. jejuni* 81-176 was naturally transformed with pEF74Cm and mutants were selected on MH-TV containing Cm after 5-7 days of growth. Mutants (designated *Δ1284*) were verified by PCR analysis with 1284-1 and 1284-2.

For complementation of *amiA*, the *amiA* gene was PCR amplified with amiA-8 (*XbaI*) and amiA-9 (*XbaI*) from *C. jejuni* 81-176 genomic DNA, digested with *XbaI* and cloned into the similarly digested pRRK integration vector (Karlyshev & Wren, 2005). The resulting construct (pEF49F) was verified for orientation by PCR with the *amiA* gene in the same orientation as the antibiotic resistance cassette encoded by the vector, and sequenced. Plasmids were inserted into *C. jejuni* wild type 81-176 and *ΔamiA* by natural transformation and transformants selected on the appropriate antibiotics. Single insertions into the rRNA spacer region were verified by PCR with primers ak233, ak234, ak235 (Karlyshev & Wren, 2005) and aphA3-2 for pRRK. Wild type phenotypes were only restored when the *amiA* gene was deleted in a wild type strain carrying the second copy of *amiA* at the rRNA locus and not in an *ΔamiA* strain into which the *amiA* gene had been inserted. To knockout *amiA* in 81-176+*amiA*, this strain was naturally

transformed with both *ΔamiA* genomic DNA and with pEF18Cm and mutants were selected on MH-TV containing Km and Cm. PCR and restriction enzyme analysis were used to verify deletion of *amiA* and the presence of *amiA* at the rRNA locus (this strain was designated *ΔamiA-c*).

A double mutant in *ΔamiA* and *Δpgp1* was constructed by deleting *ΔamiA* in the *Δpgp1* strain. The *Δpgp1* mutant strain was transformed with both *ΔamiA* genomic DNA and with pEF18Cm and mutants designated *Δpgp1 ΔamiA* were selected on MH-TV containing Km and Cm. PCR analysis was used to verify deletion of *amiA*.

Bacterial strains and plasmids

Strain or Plasmid	Genotype, serotype or description	Reference or Source
C. jejuni		
81-176	Wild type isolated from a diarrheic patient	(Korlath *et al.*, 1985)
Δ*pgp1*	81-176 *pgp1*::*aphA3*; Km^R	(Frirdich *et al.*, 2012)
Δ*pgp2*	81-176 Δ*pgp2*	(Frirdich *et al.*, 2014)
Δ*pgp1*Δ*pgp2*	81-176 *pgp1*::*aphA3*Δ*pgp2*	(Frirdich *et al.*, 2014)
Δ*pgp1-c*	81-176 Δ*pgp1 rrn::pgp1*; Km^R Cm^R	(Frirdich *et al.*, 2012)
Δ*pgp2-c*	81-176 Δ*pgp2 rrn::pgp2*	(Frirdich *et al.*, 2014)
81-176+*pgp1*	81-176 *rrn::pgp1* (from pEF20); Km^R	(Frirdich *et al.*, 2012)
Δ*amiA*	81-176 *amiA*::*cat*; Cm^R	This study
Δ*1284*	81-176 *1284*::*aphA3*; Km^R	This study
Δ*pgp1*Δ*amiA*	81-176 *pgp1*::*aphA3 amiA*::*cat*; Km^R Cm^R	This study
81-176+*amiA*	81-176 *rrn::amiA* (from pEF49F); Km^R	(Frirdich *et al.*, 2012)
Δ*amiA*-c	81-176 Δ*amiA rrn::amiA* (from pEF49F); Km^R Cm^R	This study
81-176 (pRY112-$P_{atpF'}$-*gfp*)	Wild type 81-176 strain expressing plasmid-encoded GFP from *the C. jejuni atpF'* promoter; Cm^R	(Apel *et al.*, 2012)
Δ*pgp1*(pRY112-$P_{atpF'}$-*gfp*)	Wild type 81-176 strain expressing plasmid-encoded GFP from the C. jejuni atpF' promoter; Km^R Cm^R	This study
E. coli		
DH5α	F^-, ϕ80d *deoR lacZ*Δ*M15 endA1 recA1 hsdR17*(r_K-m_K+) *supE44 thi-1 gyrA96 relA1* Δ*(lacZYA-argF) U169*	Invitrogen
**Plasmids**		
pGEM-T	PCR cloning vector; Ap^R	Promega
pUC18-K2	Source of non-polar *aphA3* cassette; Ap^RKm^R	(Menard *et al.*, 1993)
pRY109	Source of *cat* cassette; Ap^RCm^R	(Yao *et al.*, 1993)
pRRC	*C. jejuni* rRNA spacer integration vector; Cm^R	(Karlyshev & Wren, 2005)
pRRK	*C. jejuni* rRNA spacer integration vector; Km^R	J. Ketley

pRY112-$P_{atpF'}$-*gfp*	C. jejuni replicating plasmid pRY112 derivative expressing GFP from the C. jejuni atpF' promoter; Cm^R	(Apel *et al.*, 2012)
pEF18	pGEM-T ligated to *amiA* amplified with primers amiA-1 and amiA-2; Ap^R	This study
pEF18Cm	pGEMT-*amiA* (pEF18) with the *amiA* gene disrupted with the *cat* cassette; Ap^RCm^R	This study
pEF74	pGEM-T ligated to *1284* amplified with primers 1284-1 and 1284-2; Ap^R	This study
pEF74Cm	pGEMT-*1284* (pEF74) with the *1284* gene disrupted with the *cat* cassette; Ap^RCm^R	This study
pEF49F	pRRK derivative coding for amiA amplified with amiA-8 and amiA-9; Km^R	This study

Primers

Primer	Sequence 5′ to 3′	Restriction Site	Reference
ak233	GCAAGAGTTTTGCTTATGTTAGCAC		(Karlyshev & Wren, 2005)
ak234	GAAATGGGCAGAGTGTATTCTCCG		(Karlyshev & Wren, 2005)
ak235	GTGCGGATAATGTTGTTTCTG		(Karlyshev & Wren, 2005)
cat-2	GTTTTTTGGATGAATTACAAGA		(Frirdich *et al.*, 2012)
aphA3-2	CTATTTTTTGACTTACTGGGGA		(Frirdich *et al.*, 2012)
amiA-1	ATGAACAGTGCTTAGATCCAAATTATCAG		This study
amiA-2	AGAAAGTCTTGCCACTTCAAAGATT		This study
amiA-3	ATGAAATTTTACTgGTACcTCACTTTTAGAACC	*KpnI*	This study
amiA-5	CGTAGATGGCGGTGTAAGAG	*XbaI*	This study
amiA-6	TGGCTTTAGTGCCTATCATTTCATCT		This study
amiA-7	CTTGATATACCCGCACCAATAATAGCTA		This study
amiA-8	ATGAACAGTGtcTAGATCCAAATTATCAG	*XbaI*	This study
amiA-9	AGGAGTATTATCtagAAAGATTAGCTTAGC	*XbaI*	This study
1284-1	GAGCGGATTTGTTTATTTCTATACATGC		This study
1284-2	CGTTGGAGTATCACTTCTTAACAAACT		This study
1284-3	GGGGTACCGGAGTATTATCCTTAAAGATTAGCTTAGCTTTTTT		This study
1284-4	GCAAGGTATTTGTGTGCATTGATAAA	*XbaI*	This study

1344-QRT-F	ATTCAAGGTGATGAGCCTGGTGGA	This study
1344-QRT-R	TTCCCACGCGAACGCTTGATAATG	This study
rpoA-QRT-F	CGAGCTTGCTTTGATGAGTG	(Apel *et al.*, 2012)
rpoA-QRT-R	AGTTCCCACAGGAAAACCTA	(Apel *et al.*, 2012)

Whole-genome sequencing

Genomic DNA for all experiments was harvested via Wizard genomic DNA purification (Promega). Illumina libraries were prepared using the KAPA Low-Throughput Library Preparation Kit with Standard PCR Amplification Module (Kapa Biosystems, Wilmington, MA), following manufacturer's instructions except for the following changes: 750ng DNA was sheared using an M220 instrument (Covaris, Woburn, MA) in 50ul screwcap microtubes at 50 peak power, 20 duty factor, 20°C, 200 cycles per burst and 25 seconds duration. Adapter ligated fragments were size selected to 700-800bp following Illumina protocols. Standard desalting TruSeq LT and PCR Primers were ordered from Integrated DNA Technologies (Coralville, IA) and used at 0.375 µM and 0.5 µM final concentrations, respectively. PCR was reduced to 4 cycles. Libraries were quantified using the KAPA Library Quantification Kit (Kapa), except with 10 µl volume and 90 sec annealing/extension PCR, then pooled and normalized to 4 nM. Pooled libraries were re-quantified by ddPCR on a QX200 system (Bio-Rad), using the Illumina TruSeq ddPCR Library Quantification Kit and following manufacturer's protocols, except with an extended 2 min annealing/extension time. The libraries were sequenced 2x250 bp paired end v2 on a MiSeq instrument (Illumina) at 13.5 pM, following manufacturer's protocols. The MiSeq reads were reference assembled to the genome of the background strains *C. jejuni* 81-76 (NC_008787) or NCTC 11168 (AL11116) using Geneious 9.1 reference assembler (Biomatters, Auckland, NZ).

Table S1. The mean percentage and standard error of helical, coccoid and cells transitioning to the coccoid form in *C. jejuni* wild type 81-176, Δ*pgp1* mutant strain, Δ*pgp1* complemented strain (Δ*pgp1*-c) and *pgp1* overexpressing strain (81-176+*pgp1*) grown on solid media at 38 °C over 8 days (depicted graphically in Fig. 1B); *C. jejuni* wild type 81-176 grown in liquid culture at 38 °C at 4 days; and *C. jejuni* wild type 81-176 on solid media incubated at 4 °C for 29 days after an initial day of growth at 38 °C. At least three separate fields of view of approximately 200 bacteria/field of view were counted for each strain at each timepoint. Representative cells considered to be helical, coccoid or transitioning to the coccoid form are indicated by **a**, **b** or **c**, respectively in the DIC images in Fig. 1A.

		**Helical/Straight**		**Coccoid**		**Transitioning to coccoid**	
		**Mean**	**SEM**	**Mean**	**SEM**	**Mean**	**SEM**
**Day 1**	81-176	98.4	1.0	1.4	0.8	0.2	0.2
	Δ*pgp1*	100.0	0.0	0.0	0.0	0.0	0.0
	Δ*pgp1*-c	98.9	0.9	0.7	0.5	0.4	0.4
	81-176 + *pgp1*	97.8	0.8	1.3	0.2	0.9	0.6
	Δ*amiA*	98.1	0.5	1.6	0.5	0.3	0.0
	Δ*amiA* Δ*pgp1*	100.0	0.0	0.0	0.0	0.0	0.0
	81-176	95.8	3.6	3.4	3.4	0.8	0.41
	Δ*pgp2*	99.2	0.8	0.8	0.8	0.0	0.0
	Δ*pgp2*-c	100.0	0.0	0.0	0.0	0.0	0.0
	Δ*pgp1*Δ*pgp2*	100.0	0.0	0.0	0.0	0.0	0.0
**Day 2**	81-176	1.1	0.4	98.4	0.3	0.5	0.3
	Δ*pgp1*	45.3	5.9	49.7	6.4	5.0	1.8
	Δ*pgp1*-c	15.6	2.5	78.3	2.0	6.1	0.5
	81-176 + *pgp1*	4.6	2.0	94.9	2.3	0.5	0.3
	81-176	1.4	0.3	97.1	0.6	1.5	0.6
	Δ*pgp2*	4.0	2.8	94.9	2.9	1.1	0.3
	Δ*pgp2*-c	2.8	1.0	95.1	1.2	2.1	0.3
	Δ*pgp1*Δ*pgp2*	3.8	0.5	95.5	0.8	0.8	0.4
**Day 3**	81-176	1.2	0.6	97.6	0.8	1.2	0.5
	Δ*pgp1*	16.3	1.9	79.9	2.3	3.8	0.7
	Δ*pgp1*-c	1.7	0.6	97.0	0.8	1.4	0.8
	81-176 + *pgp1*	4.2	0.3	95.0	0.7	0.8	0.4
	81-176	2.3	0.7	96.5	1.2	1.2	0.7
	Δ*pgp2*	2.2	0.6	97.2	0.4	0.6	0.2
	Δ*pgp2*-c	2.3	0.3	96.3	0.9	1.4	0.6
	Δ*pgp1*Δ*pgp2*	3.5	0.8	96.4	0.7	0.1	0.1
**Day 4**	81-176	1.4	0.7	97.8	0.6	0.8	0.3
	Δ*pgp1*	14.5	5.1	83.1	4.8	2.4	1.2
	Δ*pgp1*-c	3.7	0.5	95.9	0.7	0.4	0.2
	81-176 + *pgp1*	2.3	1.2	95.8	1.3	1.9	0.2
	Δ*amiA*	45.7	0.3	48.6	0.4	5.6	0.2
	Δ*amiA* Δ*pgp1*	91.9	0.5	7.3	0.4	0.8	0.1
	81-176	1.3	0.6	96.3	0.9	2.5	1.0

	Δ*pgp2*	1.2	0.2	98.2	0.3	0.6	0.4
	Δ*pgp2*-c	0.9	0.3	96.9	0.8	2.2	0.8
	Δ*pgp1*Δ*pgp2*	2.7	0.6	95.5	1.4	1.8	0.8
	81-176 (broth)	3.1	0.3	90.6	1.6	6.1	1.9
	81-176pGFP (broth)	3.0	1.8	92.7	1.8	4.4	0.1
**Day 8**	81-176	0.4	0.2	97.6	0.4	2.0	0.6
	Δ*pgp1*	9.8	1.2	89.0	1.2	1.2	0.2
	Δ*pgp1*-c	1.2	0.7	97.9	0.2	1.0	0.5
	81-176 + *pgp1*	2.2	0.5	96.4	1.1	1.3	1.1
	Δ*amiA*	36.7	0.2	55.3	0.4	8.0	0.3
	Δ*amiA* Δ*pgp1*	86.0	0.3	13.0	0.3	1.0	0.2
	81-176	1.1	0.6	96.4	1.2	2.5	0.7
	Δ*pgp2*	1.4	0.6	96.1	2.6	2.5	2.0
	Δ*pgp2*-c	0.6	0.2	97.9	1.3	1.6	1.3
	Δ*pgp1*Δ*pgp2*	1.9	0.2	97.2	0.5	1.0	0.3
**Day 29**	81-176 (grown at 4 C)	4.3	0.6	88.7	1.6	7.0	1.0

Table S2. Genomic changes relative to the *C. jejuni* 81-176 reference sequence in GenBank (CP000538) of our laboratory strain of *C. jejuni* 81-176 (denoted 81-176 in the table; helical morphology), Δ*amiA* mutant strains (EF262 and EF280; morphology consisting of helical chains of cells with growth defect) and Δ*amiA* suppressor strains generated through laboratory passage (EF262-P5 and EF280-P5, both passaged 5 times, and EF188 with an unknown amount of passages; morphology consisting of shorter chains of cells and wild type growth). The *cat*^{R} resistance cassette insertion in *amiA* is not shown.

									Sample[1]					
**No.**[2]	**Locus**	**Putative function**	**Nucleotide position**	**Change**	**Codon change**	**Amino acid change**	**Polymorphism**	**Protein effect**	**81-176**	**Δ*amiA* EF262**	**Δ*amiA*-P5 EF262-P5**	**Δ*amiA* EF280**	**Δ*amiA*-P5 EF280-P5**	**Δ*amiA* EF188 (unknown number of passages)**
1*		Intergenic between hypothetical proteins CJJ81176_0082 and CJJ81176_0083	76 411	(C)9 -> (C)10			Insertion (tandem repeat)					88.9%	92.9%	
2*	CJJ81176_0113	LctP; L-lactate permease	99 044	(T)7 -> (T)8			Insertion (tandem repeat)	Frame Shift		92.9%	85.20%	88.60%	94.7%	100%
3*	CJJ81176_0206	Conserved hypothetical protein	178 080	C -> G	ACC -> ACG		Insertion (tandem repeat)	Frame Shift					85.0%	
4*	CJJ81176_0227	PurF; amidophosphoribosyltransferase	197 289	C -> T	AGC -> AAC	S -> N	SNP (transition)	Substitution		100%	100%	100%	100%	100%
5	CJJ81176_0716	MraW; S-adenosyl-methyltransferase	646 040	(T)5 -> (T)4			Deletion (tandem repeat)	Frame Shift		97.4%				
6	CJJ81176_0716	MraW; S-adenosyl-methyltransferase	646 114	C -> A			SNP (transversion)	Truncation						100%
7*	CJJ81176_0758	Conserved hypothetical protein	685 536	(G)9 -> (G)8			Deletion (tandem repeat)	Frame Shift					92.9%	
8		Intergenic, between CJJ81176_0765 and CJJ81176_1724 (rrsC, 16S ribosomal RNA)	692 439	(CC)5 -> (CC)4			Deletion (tandem repeat)				100%			
9*		Intergenic, between CJJ81176_0765 and CJJ81176_1724 (rrsC, 16S ribosomal RNA)	692 440	(C)11 -> (C)10			Deletion (tandem repeat)		88.6%	80.0%	89.50%		82.40%	
10*		Intergenic between CJJ81176_0920 (CysK, cysteine synthase A) and CJJ81176_1731 (Hup, DNA-binding protein HU)	849 698	+T			Insertion		100%	100%	95.70%	100%	100%	
11*		Intergenic between CJJ81176_0920 (CysK, cysteine synthase A) and CJJ81176_1731 (Hup, DNA-binding protein HU)	849 704	(T)2 -> (T)3			Insertion (tandem repeat)		96.3%	96.6%	95.8%	96.9%	100%	
12*	CJJ81176_1105	Peptidase, M23/M37 family	1 021 802	T -> C	AGC -> GGC	S -> G	SNP (transition)	Substitution	100%	100%	100%	100%	100%	100%
13*	CJJ81176_1160	Beta-1,4-N-acetylgalactosaminyltransferase	1 079 106	(G)10 -> (G)9			Deletion (tandem repeat)	Frame Shift	90.9%	93.3%	93.3%	100%	100%	100%
14*	CJJ81176_1354	Enterochelin ABC transporter, periplasmic enterochelin-binding protein	1 271 069	A -> G	AAT -> GAT	N -> D	SNP (transition)	Substitution	100%	100%	100%	100%	100%	100%
15	CJJ81176_1419	Putative methyltransferase	1 341 403	(C)9 -> (C)10			Insertion (tandem repeat)	Frame Shift					84.6%	85.0%

[1]The percentages shown represent the variant frequency. A blank cell indicates no change in that strain in comparison to the published sequence.
[2]An asterisk (*) represents a mutation previously identified in our wild type 81-176 and mutant strains unrelated to Δ*amiA* by whole genome sequencing and likely represent hypervariable regions in the *C. jejuni* genome (Frirdich *et al.*, 2017).

Table S3. Legend for strain designations in Table 1, 2 and Table S4.

Series	Date Analyzed	Strain number	Strain description
A	09/2010	1	Δ*pgp1* Day 1
		2	81-176+*pgp1* Day 1
B	01/2011	1	81-176 Day 1
		2	81-176 Day 4
		3	Δ*pgp1* Day 4
		4	Δ*pgp2* Day 1
C	06/2011	1	Δ*amiA*Δ*pgp1* Day 1
D	12/2011	1	81-176 Day 1
		2	Δ*amiA* Day 1
E	01/2013	1	81-176 Day 1
		2	81-176 4 °C Day 29
F	08/2013	1	81-176 Day 1
		2	81-176 Day 4
		3	Δ*amiA* Day 4
		4	81-176+*amiA* Day 1
		5	Δ*amiA*Δ*pgp1* Day 4
G	09/2014	1	Δ*pgp2* Day 4
		2	81-176+*pgp1* Day 4

Table S4A. Muropeptide composition of the wild-type 81-176, Δ*pgp1*, 81-176+*pgp1* (*pgp1* overexpressing strain), and Δ*pgp2* strains grown for either 1 or 4 days at 38 °C (strains were grown at 38 °C unless otherwise indicated) or 29 days at 4 °C summarized in Table 1.

		***C. jejuni* strains**											
		**81-176 Day 1**	**81-176 Day 4**	**81-176 Day 1**	**81-176 Day 4**	**81-176 Day 1**	**81-176 4 °C Day 29**	**Δ*pgp1* Day 1**	**Δ*pgp1* Day 4**	**Δ*pgp2* Day 1**	**Δ*pgp2* Day 4**	**81-176 +*pgp1* Day 1**	**81-176 +*pgp1* Day 4**
	**Strain designation[1]**	B-1	B-2	F-1	F-2	E-1	E-2	A-1	B-3	B-4	G-1	A-2	G-2
**Peak number**	**Muropeptide species**	**% Peak area**											
1	Tri	7.29	3.59	8.75	3.77	5.83	4.86	27.1	31.69	0.00	0.00	1.9	0.97
2	Tetra	15.70	11.33	0.41	0.00	16.10	15.29	2.7	1.28	29.31	18.17	18.6	9.03
	PentaGly5	nd[3]	nd	16.06	10.93	0.56	0.28	nd	nd	nd	0.78	nd	0.67
3	Di	13.52	23.64	0.82	0.44	16.25	20.62	4.2	7.96	8.14	17.41	15.4	28.21
4	Tri-Ac [2]	1.13	0.28	12.96	23.17	0.32	0.36	3.5	0.18	0.00	0.70	0.3	0.28
	Penta	nd	nd	1.01	0.39	0.00	0.26	nd	nd	nd	nd	nd	nd
5	Tetra-Ac [2]	0.66	0.00	0.61	0.00	0.61	0.81	0.0	0.00	0.96	0.00	1.6	0.00
6	Di-Ac	0.90	0.30	0.24	0.66	0.00	0.22	1.0	0.27	0.52	0.00	1.9	0.56
7	TetraTri	9.70	6.92	10.53	7.05	9.72	10.44	20.9	23.51	0.00	0.35	2.4	2.87
8	TetraPentaGly5	0.69	0.95	0.77	0.77	0.39	0.48	0.7	0.48	0.77	1.39	1.8	1.73
9	TetraTetra	20.74	22.19	21.40	22.25	19.01	20.25	8.7	6.60	32.69	28.71	27.8	23.57
	TetraPenta	nd	nd	nd	nd	0.00	0.24	nd	nd	nd	nd	nd	nd
10	TetraTri-Ac [2]	1.94	0.24	1.47	0.44	1.78	1.33	4.7	0.42	0.00	0.00	0.3	0.00
11	TetraTetraTri	0.96	1.09	0.96	1.01	0.78	0.77	1.7	2.11	0.00	0.39	0.3	0.62
12	TetraTetra-Ac [2]	0.31	0.17	1.65	0.64	0.25	0.39	1.4	0.00	0.43	0.00	4.9	0.28
13	TetraTetraTetra	4.29	4.35	2.54	3.46	2.93	3.87	0.6	0.87	5.56	4.71	2.4	4.90
14	TetraTriAnh I	0.89	0.58	1.10	0.97	1.15	1.13	2.4	2.97	0.00	0.00	0.4	0.20
15	TetraTriAnh II	2.59	1.70	2.36	1.86	2.63	2.19	5.0	7.49	0.00	0.00	0.3	0.49
16	TetraTetraAnh I	3.04	3.68	2.77	3.16	3.00	2.88	1.5	1.20	4.63	5.32	4.9	3.19
17	TetraTetraAnh II	5.26	5.58	5.20	6.20	5.56	4.99	1.5	1.72	6.96	6.60	4.3	4.94
	TetraTetraTriAnh	nd	nd	1.13	1.17	nd	nd	nd	nd	nd	0.00	nd	0.80
18	TetraTetraTetraAnh	4.63	5.94	4.06	5.71	4.33	3.41	1.5	1.37	5.52	6.42	4.8	6.87
	All known	94.24	92.53	96.80	94.05	91.20	95.07	88.9	90.12	95.49	90.95	94.1	90.18

[1] The strain designation consists of a letter denoting the series in which the sample was analyzed followed by a number denoting the sample within the series. Samples analyzed in the same batch will have identical series letters.

[2] The values for the percentage of O-acetylated species do not represent the true level of O-acetylation in these strains, as most of these substitutions are lost in the standard alkaline reduction procedure used in this study to prepare the PG. These values were included to demonstrate the relative difference in O-acetylation between the samples, but actual comparisons between the samples were not made.

[3] nd = not determined.

Table S4B. Muropeptide composition of the *C. jejuni* Δ*amiA*, 81-176+*amiA* (*amiA* overexpressing strain), and Δ*amiA*Δ*pgp1* strains grown for either 1 or 4 days at 38 °C summarized in Table 2 (the muropeptide composition of the additional strains shown in Table 2 are reported in Table S2A).

		***C. jejuni* strains**				
		**Δ*amiA* Day 1**	**81-176+*amiA* Day 1**	**Δ*amiA*Δ*pgp1* Day 1**	**Δ*amiA* Day 4**	**Δ*amiA*Δ*pgp1* Day 4**
	**Strain designation [1]**	D-2	F-4	C-1	F-3	F-5
**Peak number**	**Muropeptide species**	**% Peak area**				
1	Tri	8.15	6.98	21.98	6.54	23.63
2	Tetra	16.29	14.73	3.80	4.97	0.92
	PentaGly5	nd[3]	0.99	nd	0.00	0.00
3	Di	14.18	15.39	8.58	28.02	15.97
4	Tri-Ac [2]	0.35	1.25	3.10	0.70	0.68
	Penta	nd	nd	nd	nd	nd
5	Tetra-Ac [2]	0.07	0.87	0.13	0.23	0.00
6	Di-Ac	0.13	1.10	1.56	1.12	0.68
7	TetraTri	11.48	9.29	17.87	10.86	23.87
8	TetraPentaGly5	0.68	1.12	1.21	0.74	0.47
9	TetraTetra	22.26	20.42	10.25	20.07	8.67
	TetraPenta	nd	nd	nd	nd	nd
10	TetraTri-Ac [2]	0.18	1.70	4.58	1.13	0.90
11	TetraTetraTri	1.10	1.28	1.67	1.16	2.31
12	TetraTetra-Ac [2]	0.08	2.16	1.70	1.49	0.35
13	TetraTetraTetra	3.04	2.50	1.03	2.57	1.16
14	TetraTriAnh I	1.01	1.03	1.89	1.22	2.63
15	TetraTriAnh II	2.17	2.43	3.88	2.36	6.71
16	TetraTetraAnh I	2.85	2.72	1.84	2.65	1.32
17	TetraTetraAnh II	5.15	5.38	1.76	4.96	2.10
	TetraTetraTriAnh	nd	1.06	nd	0.99	3.54
18	TetraTetraTetraAnh	4.29	4.12	2.23	4.04	1.97
	All known	93.46	96.89	89.06	95.82	97.88

[1] The strain designation consists of a letter denoting the series in which the sample was analyzed followed by a number denoting the sample within the series. Samples analyzed in the same batch will have identical series letters.
[2] The values for the percentage of O-acetylated species do not represent the true level of O-acetylation in these strains, as most of these substitutions are lost in the standard alkaline reduction procedure used in this study to prepare the PG. These values were included to demonstrate the relative difference in O-acetylation between the samples, but actual comparisons between the samples were not made.
[3] nd = not determined.

Table S5. Comparison of the muropeptide dipeptides, tripeptides and tetrapeptides (from Tables 1 & 2) of the *C. jejuni* wild-type 81-176, Δ*pgp2*, 81-176+*pgp1* (pgp1 overexpressing strain), Δ*pgp1*, Δ*amiA,* and Δ*amiA*Δ*pgp1* strains grown for 1 and 4 days at 38 °C with varying amounts of coccoid cells present in the population. The percent change between Day 1 and Day 4 of the muropeptides of the same strain are shown below the Day 4 values.

	C. jejuni strains											
	**81-176 Day 1**	**81-176 Day 4**	**Δ*pgp2* Day 1**	**Δ*pgp2* Day 4**	**81-176 +*pgp1* Day 1**	**81-176 +*pgp1* Day 4**	**Δ*pgp1* Day 1**	**Δ*pgp1* Day 4**	**Δ*amiA* Day 1**	**Δ*amiA* Day 4**	**Δ*amiA* Δ*pgp1* Day 1**	**Δ*amiA* Δ*pgp1* Day 4**
**Shape**	Helical	Coccoid (97.8%)	Straight	Coccoid (98.2%)	Straight	Coccoid (95.8%)	Straight	Rods & coccoids (83% coccoid)	Helical chains	Helical chains & coccoids (48.6% coccoid)	Straight chains	Straight chains (7.3% coccoid)
**Strain designation[1]**	B-1	B-2	B-4	G-1	A-2	G-2	A-1	B-3	D-2	F-2	C-1	F-5
**Strain used for comparison**		B-1		B-4		A-2		A-1		D-2		C-1
**Muropeptide species**	% Peak area											
Dipeptides (Total)	15.3	25.9	9.1	19.1	18.4	31.9	5.9	9.1	15.3	30.4	11.4	17.0
		(+69.3%)		(+109.9%)		(+73.4%)		(+54.2%)		(+98.7%)		(+49.1%)
Monomeric tripeptides	8.9	4.2	0.0	0.8	2.3	1.4	34.4	35.4	9.1	7.6	28.2	24.8
		(-52.8%)		(+)		(-39.1%)		(+2.9%)		(-16.5%)		(-12.1%)
Monomeric tetrapeptides	17.4	12.2	31.7	19.9	21.5	10.0	3.1	1.4	17.5	5.4	4.4	0.9
		(-29.9%)		(-51.1%)		(-53.4%)		(-54.8%)		(-69.1%)		(-79.5%)
Tripeptides (Total)	17.3	9.7	0.0	1.1	4.2	3.6	53.6	55.2	17.4	16.1	44.6	43.1
		(-43.9%)		(+)		(-14.3%)		(+3.0%)		(-7.5%)		(-3.4%)
Tetrapeptides (Total)	67.0	63.9	90.5	78.1	76.5	61.9	40.2	35.4	66.9	52.1	43.3	36.1
		(-4.6%)		(-13.7%)		(-19.1%)		(-11.9%)		(-22.1%)		(-16.6%)

[1] The strain designation consists of a letter denoting the series in which the sample was analyzed followed by a number denoting the sample within the series. Samples analyzed in the same batch will have identical series letters.

Table S6. Comparison of the muropeptide dipeptides, tripeptides, tetrapeptides, and anhydropeptides of *H. pylori* 26695 and *ΔamiA* strain grown for 8 h and 48 h published previously (Chaput *et al.*, 2016). The percent change between 8 h and 48 h of the muropeptides of the same strain are shown below the 48 h values.

	***H. pylori* strains**			
	**26695 8 h**	**26695 48 h**	***ΔamiA* 8 h**	***ΔamiA* 48 h**
**Muropeptide species**	**% Peak area**			
Dipeptides (Total)	3.3	23.3 (+606.1%)	1.7	10.3 (+505.9%)
Monomeric tripeptides	16.8	4.9 (-70.2%)	13.5	14.6 (+8.1%)
Monomeric tetrapeptides	5.2	2.6 (-50.0%)	6.7	3.8 (-43.2%)
Tripeptides (Total)	25.2	14.1 (-44.0%)	19.7	21.8 (+10.7%)
Tetrapeptides (Total)	41.8	40.6 (-2.9%)	40.9	34.4 (-15.9%)
Anhydropeptides (Total)	13.0	15.8 (+21.5%)	12.2	6.7 (-45.1%)

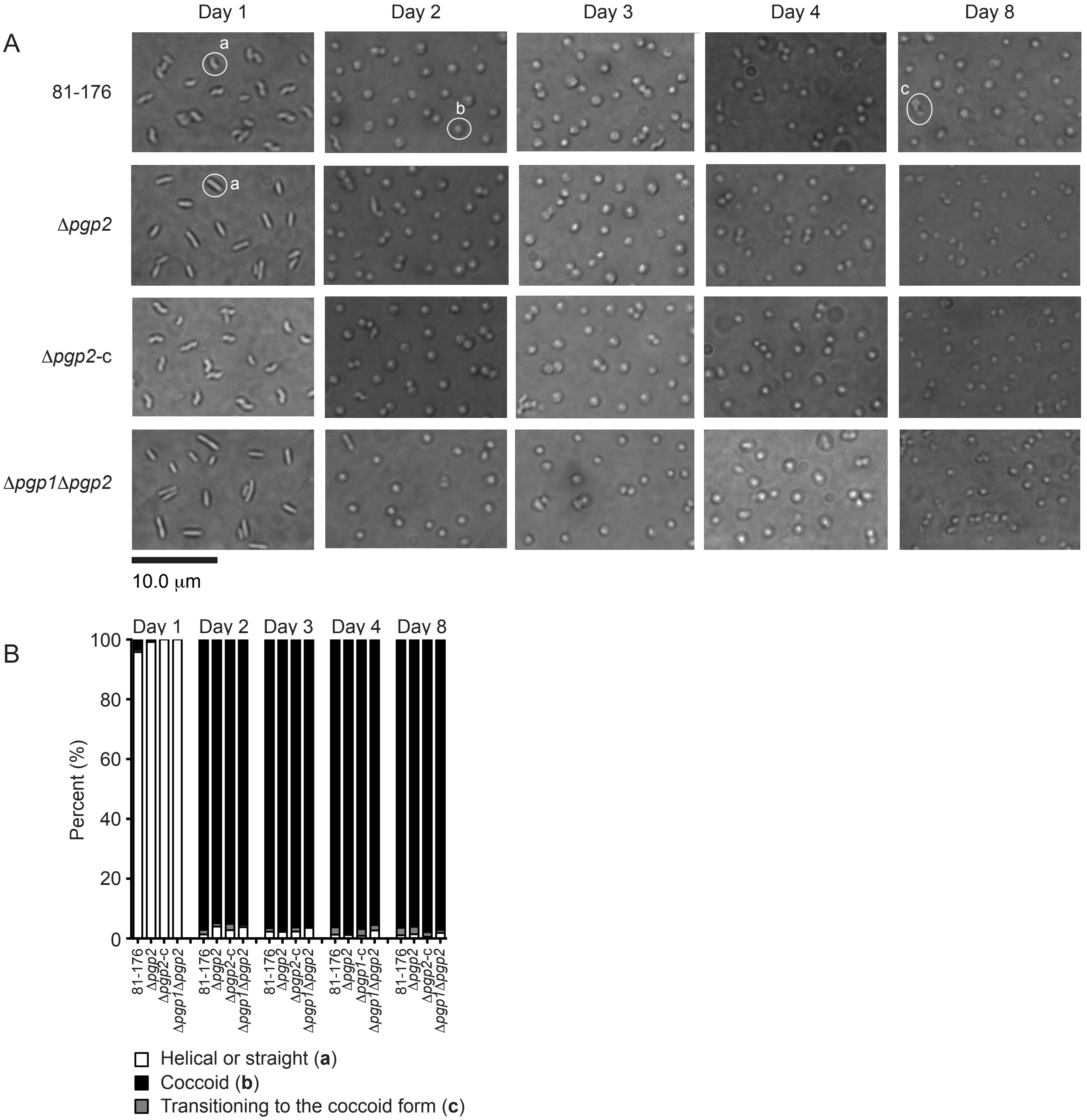

Figure S1. The *C. jejuni* transition of Δ*pgp2* and Δ*pgp1*Δ*pgp2* mutant strains from a helical to coccoid form was similar to wild type. A, DIC microscope images taken over 8 days. *C. jejuni* wild type 81-176, Δ *pgp2* mutant strain, Δ*pgp2* complemented strain (Δ*pgp2-c*) and Δ*pgp1*Δ*pgp2* double mutant strain grown on solid media at 38 °C to follow the transition to the coccoid form over time. **B**, the percentage of helical, coccoid and cells transitioning to the coccoid form as determined from DIC images such as those shown in **A**. At least three separate fields of view of approximately 200 bacteria/field of view were counted for each strain at each timepoint and this was carried out in triplicate. Representative cells considered to be helical, coccoid or transitioning to the coccoid form are indicated by **a**, **b** or **c**, respectively in the DIC images in **A**. There were no statistically significant differences in coccoid formation between wild type and Δ*pgp2*, Δ*pgp2-c* or Δ*pgp1*Δ*pgp2* at each timepoint using the unpaired Student's t-test. No differences in growth characteristics were observed for any of the strains by growth curve analysis in liquid and on solid media (data not shown).

```
CJ_AmiA   MAKIFVFLVFVFTTLFGAYENELANFDKNFIGSKSEVQVKFHHQLKSLYIQSVINEDEKT   60
HP_AmiA   ------------------------------------------------------------   0
EC_AmiB   ------------------------------------------------------------   0
EC_AmiA   ------------------------------------------------------------   0
EC_AmiC   ------------------------------------------------------------   0

CJ_AmiA   KIEILKRLIISSNTLNLDDKSYANELKESGISEASINALRKAVVKDIKVQNIPQDAEKST   120
HP_AmiA   ------------------------------------------------------------   0
EC_AmiB   ------------------------------------------------------------   0
EC_AmiA   ------------------------------------------------------------   0
EC_AmiC   ------------------------------------------------------------   0

CJ_AmiA   FEPIKTVQKNEKKSSNEQIKNTEKTNSKTSQAPKSSIVSKVKSDDKIYVLKSS-----K-   174
HP_AmiA   ------------------------------------MLVRLGVVACLFWLHYAYATTLKI   24
EC_AmiB   -----------------------------------------MMYRIRNWLVATLLLLCTP   19
EC_AmiA   ------------------------------------------------------------   0
EC_AmiC   -----------------------MSGSNTAIS-----RRRLLQGAGAMWLLSVSQVSLAA   32

CJ_AmiA   --------------T-SQGVEFELNADLDKNQLKSFALDEKGNYRFISD-----FDGVLE   214
HP_AmiA   --------TNVVPFG-SSSVKMVFNQ-----EVKKFKEVSLKNFKSYLE-----LEAILT   65
EC_AmiB   VGAATLSDIQVSNGNQQARITLSFI------GDPDYAFSHQSKRTVALDIK---QTGVIQ   70
EC_AmiA   ------------------------------------------------------------   0
EC_AmiC   VSQ--VVAVRVWPASSYTRVTVESNRQL---KYKQFALSNP--ERVVVDIEDVNLNSVLK   85

CJ_AmiA   GGKKEFKFDEY----RLVISQFNPKTIRIVVYAKEKIPIDISFKEEKLIFSKETTSQKKT   270
HP_AmiA   IPKKHYQFSKQ---SFITIAQFSPKLVRVVIGYAPKMTYEVKILKDKLYVSIVEKKPLIR   122
EC_AmiB   GLPLLFSGN-NLVKAIRSGTPKDAQTLRLVVDLTENGKTEAVKRQNGS------------   117
EC_AmiA   -----------------MS-TF--------------------------------------   4
EC_AmiC   GMAAQIRADDPFIKSARVG-QFDPQTVRMVFELKQNVKPQLFAL----------------   128

CJ_AmiA   EQASKISEKKQDQK-TEIKTKVLETKKQDKKTNNEKENQESLYVLDVDKISNAVVLNLSD   329
HP_AmiA   HQMALKPPKHHALKHTTPKPAHKPIKKEAKKV----------------------------   154
EC_AmiB   ------------------------------------------------------------   117
EC_AmiA   ------------------------------------------------------------   4
EC_AmiC   ------------------------------------------------------------   128

CJ_AmiA   DLDEKEIAVFDTKDQKFRYVVSFKGVLEGNRKSFTFGQNAITVTQYNPKTVR-V------   382
HP_AmiA   --KEKT------------------------PTK--HAHSKHTHSPLNERSTK-K------   179
EC_AmiB   -----------------NYTVVF-----------TINADVPPPPPPPPVVAKRVETPAVV   149
EC_AmiA   -------------------------------------------KPLKTLTSRRQVLKAGL   21
EC_AmiC   -------------------------------------------APVAGFKER---LVMDL   142
                                                             :

CJ_AmiA   --VLSAPKEFKLLKELDNKNLTLGFYTQTTNQNASKKANQSSSKTLNTNYKSGKLVVIDA   440
HP_AmiA   ----EIPKKEIPKKEAENESKNQVF------------IAEKNDTFIKTKRKKHKKIVLDA   223
EC_AmiB   APRVSEPARNPFKTES-NRTTGVI----SSNT------VTRPAARATANTGDKIIIAIDA   198
EC_AmiA   AALTLSGM-------SQAIAKDELL-K-TSNG------HSKPK-AKKS--GGKRVVVLDP   63
EC_AmiC   YPANAQDMQDPLLALLEDYNKGDLE-KQVPPA------QSGPQ-PGKAGRDRPIVIMLDP   194
                                                        .:       : :*
```

```
CJ_AmiA   GHGGKDSGALSDKKGSLKEKDIVLSTALKLGNELKKRG-YKVLYTRSSDKFINLRDRTKY   499
HP_AmiA   GHGGKDCGAMSAN--LVCEKDIVLEVVKFLHKELKKRD-YSVLLTRDKDIYIDLVARTEL   280
EC_AmiB   GHGGQDPGAIGPG--GTREKNVTIAIARKLRTLLNDDPMFKGVLTRDGDYFISVMGRSDV   256
EC_AmiA   GHGGIDTGAIGRN--GSKEKHVVLAIAKNVRSILRNHG-IDARLTRSGDTFIPLYDRVEI   120
EC_AmiC   GHGGEDSGAVGKY--KTREKDVVLQIARRLRSLIEKEGNMKVYMTRNEDIFIPLQVRVAK   252
          **** * **:.       **.:.:  .  : . :..    .   **. * :* :  *

CJ_AmiA   ANDKRADLFISIHANAAPNATKAKSSEGVETFFLSPA--RSERSKKAAEKENQGDFEEI-   556
HP_AmiA   ANKKSADLFISVHANSIPKHST-SNAHGIETYFLSTA--RSERARKVAEQENKDDVNLM-   336
EC_AmiB   ARKQNANFLVSIHADAAPNRSA----TGASVWVLSNRRANSEMASWLEQHEKQSELLGGA   312
EC_AmiA   AHKHGADLFMSIHADGFTNPKA----AGASVFALSNRGASSAMAKYLSERENRADEVAGK   176
EC_AmiC   AQKQRADLFVSIHADAFTSRQP----SGSSVFALSTKGATSTAAKYLAQTQNASDLIGG-   307
          *..: *::::*:**:.  .        * ..: **     *  :    : ::  :

CJ_AmiA   ---------NYFSKQSILNFLNREKIVASNKLAIDVQKNILTQTRKKYK-IVDGGVREAP   606
HP_AmiA   ---------DYFSKSLFLNSLNTQRLIVSNKLAIDVQYGMLQSVRKNYPDVVDGGVREGP   387
EC_AmiB   GDVLANSQSDPYLSQAVLDLQFGHSQ----RVGYDVATSMISQLQR-IGEIHKRRPEHAS   367
EC_AmiA   KA----TDKDHLLQQVLFDLVQTDTI----KNSLTLGSHILKKIKP-VHKLHSRNTEQAA   227
EC_AmiC   VS----KSGDRYVDHTMFDMVQSLTI----ADSLKFGKAVLNKLGK-INKLHKNQVEQAG   358
                   :   .  .::             .  .   :: .       : .   ...

CJ_AmiA   FWVLVGAQMPAILIEIGYITHPNEGKRIANKAFQDLLVKGIADGVESYFYNNR-------   659
HP_AmiA   FWVLAGALMPSILIEIGYNSHAIESKRIQSKPYQKILAKGIADGIDSFFSKND-------   440
EC_AmiB   LGVLRSPDIPSVLVETGFISNNSEERLLASDDYQQQLAEAIYKGLRNYFLAHPMQSAPQG   427
EC_AmiA   FVVLKSPSVPSVLVETSFITNPEEERLLGTAAFRQKIATAIAEGVISYFHWFDNQKAHSK   287
EC_AmiC   FAVLKAPDIPSILVETAFISNVEEERKLKTATFQQEVAESILAGIKAYFADGATLARRG-   417
          : ** .  :*::*:* .: ::  * : : .  ::. :. .*  *:  :*

CJ_AmiA   ------------------ 659
HP_AmiA   ------------------ 440
EC_AmiB   ATAQTASTVTTPDRTLPN 445
EC_AmiA   KR---------------- 289
EC_AmiC   ------------------ 417
```

Figure S2. Amidase multiple sequence alignment. Clustal Omega 2.1 multiple sequence alignment of *C. jejuni* AmiA (designated CJ_AmiA; WP_011812778.1; 659 amino acids) with the *H. pylori* AmiA (HP_AmiA; YP_006934687.1; 440 amino acids), and *E. coli* K-12 AmiA (EC_AmiA; YP_490671.1; 289 amino acids), AmiB (EC_AmiB; YP_492311.1; 445 amino acids) and AmiC (EC_AmiC; YP_491022.1; 417 amino acids). AMIN domain (Amidase N-terminal domain; pfam11741) residues are indicated in green. This domain is suggested to mediate the periplasmic or extracellular targeting of bacterial proteins to the cell envelope. Residues marked in blue indicate the highly conserved Amidase_3 (pfam01520) domain. This domain is found in N-acetylmuramoyl-L-alanine amidases and is involved in cleaving the amide bond between N-acetylmuramoyl and L-amino acids in bacterial PG. Residues highlighted in red are the active site residues, with the first 3 of the 4 site residues predicted to be involved in metal binding. The active site residues are highly conserved in *C. jejuni* and *H. pylori* homologs. Conserved domains and active site residues were identified with NCBI conserved domain searches.

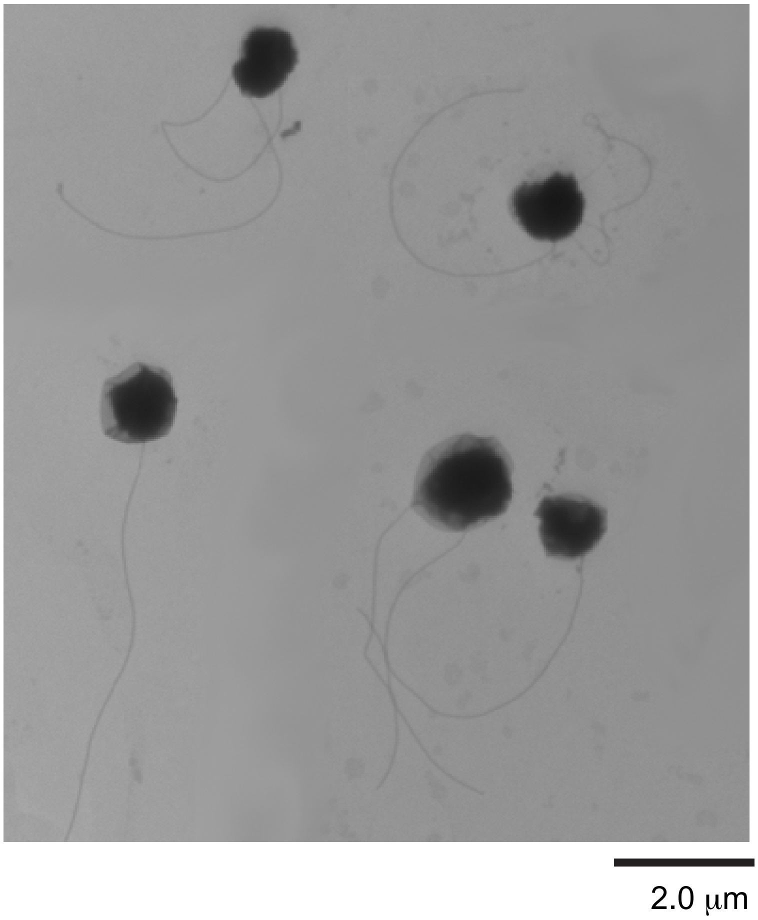

Figure S3. Coccoid *C. jejuni* retain their flagella. Negatively stained TEM images of coccoid *C. jejuni* 81-176 wild-type strain grown on plates for 48 h demonstrating the presence of flagella.

References

Apel, D., J. Ellermeier, M. Pryjma, V.J. Dirita & E.C. Gaynor, (2012) Characterization of *Campylobacter jejuni* RacRS reveals roles in the heat shock response, motility, and maintenance of cell length homogeneity. *J Bacteriol* **194**: 2342-2354.

Chaput, C., C. Ecobichon, N. Pouradier, J.C. Rousselle, A. Namane & I.G. Boneca, (2016) Role of the N-Acetylmuramoyl-l-Alanyl Amidase, AmiA, of *Helicobacter pylori* in Peptidoglycan Metabolism, Daughter Cell Separation, and Virulence. *Microb Drug Resist* **22**: 477-486.

Frirdich, E., J. Biboy, C. Adams, J. Lee, J. Ellermeier, L.D. Gielda, V.J. Dirita, S.E. Girardin, W. Vollmer & E.C. Gaynor, (2012) Peptidoglycan-modifying enzyme Pgp1 is required for helical cell shape and pathogenicity traits in *Campylobacter jejuni*. *PLoS Pathog* **8**: e1002602.

Frirdich, E., J. Biboy, S. Huynh, C.T. Parker, W. Vollmer & E.C. Gaynor, (2017) Morphology heterogeneity within a *Campylobacter jejuni* helical population: the use of calcofluor white to generate rod-shaped C. jejuni 81-176 clones and the genetic determinants responsible for differences in morphology within 11168 strains. *Mol Microbiol* **104**: 948-971.

Frirdich, E., J. Vermeulen, J. Biboy, F. Soares, M.E. Taveirne, J.G. Johnson, V.J. DiRita, S.E. Girardin, W. Vollmer & E.C. Gaynor, (2014) Peptidoglycan LD-carboxypeptidase Pgp2 influences *Campylobacter jejuni* helical cell shape and pathogenic properties and provides the substrate for the DL-carboxypeptidase Pgp1. *J Biol Chem* **289**: 8007-8018.

Karlyshev, A.V. & B.W. Wren, (2005) Development and application of an insertional system for gene delivery and expression in Campylobacter jejuni. *Appl Environ Microbiol* **71**: 4004-4013.

Korlath, J.A., M.T. Osterholm, L.A. Judy, J.C. Forfang & R.A. Robinson, (1985) A point-source outbreak of campylobacteriosis associated with consumption of raw milk. *J Infect Dis* **152**: 592-596.

Menard, R., P.J. Sansonetti & C. Parsot, (1993) Nonpolar mutagenesis of the ipa genes defines IpaB, IpaC, and IpaD as effectors of Shigella flexneri entry into epithelial cells. *J Bacteriol* **175**: 5899-5906.

Yao, R., R.A. Alm, T.J. Trust & P. Guerry, (1993) Construction of new Campylobacter cloning vectors and a new mutational cat cassette. *Gene* **130**: 127-130.
